# Admixture mapping in interspecific *Populus* hybrids identifies classes of genomic architectures for phytochemical, morphological and growth traits

**DOI:** 10.1111/nph.15930

**Published:** 2019-06-23

**Authors:** Luisa Bresadola, Céline Caseys, Stefano Castiglione, C. Alex Buerkle, Daniel Wegmann, Christian Lexer

**Affiliations:** ^1^ Department of Biology University of Fribourg Chemin du Musée 10 1700 Fribourg Switzerland; ^2^ Department of Plant Sciences University of California Davis One Shields Avenue Davis CA 95616 USA; ^3^ Department of Chemistry and Biology ‘A. Zambelli’ University of Salerno Via Giovanni Paolo II 132 84084 Fisciano, Salerno Italy; ^4^ Department of Botany University of Wyoming 1000 E. University Ave. Laramie WY 82071 USA; ^5^ Swiss Institute of Bioinformatics 1700 Fribourg Switzerland; ^6^ Department of Botany and Biodiversity Research Faculty of Life Sciences University of Vienna Rennweg 12 A‐1030 Vienna Austria

**Keywords:** admixture mapping, fitness‐related traits, genomic architecture, heritability, natural hybrids, polygenic modeling, *Populus*, RAD‐seq

## Abstract

- The genomic architecture of functionally important traits is key to understanding the maintenance of reproductive barriers and trait differences when divergent populations or species hybridize. We conducted a genome‐wide association study (GWAS) to study trait architecture in natural hybrids of two ecologically divergent *Populus* species.
- We genotyped 472 seedlings from a natural hybrid zone of *Populus alba* and *Populus tremula* for genome‐wide markers from reduced representation sequencing, phenotyped the plants in common gardens for 46 phytochemical (phenylpropanoid), morphological and growth traits, and used a Bayesian polygenic model for mapping.
- We detected three classes of genomic architectures: traits with finite, detectable associations of genetic loci with phenotypic variation in addition to highly polygenic heritability; traits with indications for polygenic heritability only; and traits with no detectable heritability. For the first class, we identified genome regions with plausible candidate genes for phenylpropanoid biosynthesis or its regulation, including MYB transcription factors and glycosyl transferases.
- GWAS in natural, recombinant hybrids represent a promising step towards resolving the genomic architecture of phenotypic traits in long‐lived species. This facilitates the fine‐mapping and subsequent functional characterization of genes and networks causing differences in hybrid performance and fitness.

## Introduction

Understanding the genetic architecture of phenotypic trait differences between divergent populations and species has long been a fundamental goal in evolutionary genetics. At the within‐species level, interest has primarily been on understanding local adaptation in wild species and the selective forces operating during domestication of agriculturally important species (Atwell *et al*., 2010; Huang *et al*., 2011; Jones *et al*., 2012; Li *et al*., 2012; Evans *et al*., 2014). At the between‐species level, evolutionary geneticists have sought to understand the origin and maintenance of adaptive trait differences and reproductive barriers between species, and thus the mechanisms maintaining species integrity (Coyne & Orr, 2004; Feder *et al*., 2012; Lindtke *et al*., 2013; Turner & Harr, 2014).

The genetic architecture of traits is frequently inferred from family‐based association studies or experimental crosses (e.g. Tanksley, 1993; Kong *et al*., 2013; Liller *et al*., 2017), but both methods are limited to organisms that exhibit short generation times, are readily crossed in the glasshouse or laboratory, and produce abundant offspring to yield sufficient power for mapping (e.g. Bradshaw *et al*., 1998; Rieseberg *et al*., 2003; Zhu *et al*., 2003). One way to extend genetic mapping to longer‐lived organisms is to use recombinants from natural hybrid zones (Barton & Hewitt, 1985; Rieseberg & Buerkle, 2002; Buerkle & Lexer, 2008). This approach, known as admixture mapping, was originally introduced by human geneticists (Chakraborty & Weiss, 1988) and its power, potential and limitations have been discussed elsewhere (e.g. Briscoe *et al*., 1994; Buerkle & Lexer, 2008; Lindtke *et al*., 2013). Briefly, the approach potentially facilitates mapping by making use of natural hybrid crosses in situations where carrying out experimental crosses would be difficult (e.g. in long‐lived species), with the power to detect associations depending largely on the lengths of haplotype blocks and thus the admixture history of populations. Despite its frequent use in human medical genetics, admixture mapping has only rarely been applied to plant and animal species (sunflowers (Rieseberg *et al*., 1999), sticklebacks (Malek *et al*., 2012), poplars (Lindtke *et al*., 2013; Suarez‐Gonzalez *et al*., 2018), canids (vonHoldt *et al*., 2016) and warblers (Brelsford *et al*., 2017)).

What makes admixture mapping attractive is the opportunity to analyze the genomic architecture of trait differences that vary between divergent populations or species. Also, compared with mapping in controlled crosses, much more of the phenotypic and genetic variation of wild species may be captured (Lexer *et al*., 2004; Buerkle & Lexer, 2008). A challenging aspect, however, is the complexity of linkage disequilibrium (LD) along the genome, which may be affected by genomic incompatibilities and coupling effects expected in hybrid zones of highly divergent populations (Barton & Hewitt, 1985; Bierne *et al*., 2011; Lindtke *et al*., 2013; Gompert *et al*., 2017).

Hybrid zones formed by *Populus* species represent textbook examples of natural interspecific crosses (Stettler *et al*., 1996; Arnold & Kunte, 2017). *Populus* is a model genus for studies of tree form, function and evolution of forest foundation species, including their involvement in eco‐evolutionary dynamics (Tuskan *et al*., 2006; Whitham *et al*., 2006). This study is focused on the ecologically divergent *Populus alba* (white poplar), widespread in southern Eurasia and northern Africa, and *Populus tremula* (European aspen), found mainly in northern Eurasia. Even after > 2.8 Myr of divergence (Christe *et al*., 2017), the reproductive barriers between these species are incomplete, and thus they hybridize in regions where their ranges overlap (Christe *et al*., 2016; Macaya‐Sanz *et al*., 2016; Zeng *et al*., 2016). Despite strong postzygotic barriers, a broad range of recombinant hybrid seeds are formed in these hybrid zones (Lindtke *et al*., 2014; Christe *et al*., 2016). Here, we use a recombinant mapping population composed of plants grown from seeds collected from open pollinated trees in a natural hybrid zone and cultivated in two common gardens. Using these, we study a range of functionally and ecologically relevant traits exhibiting phenotypic differentiation among the parental species and their hybrids, including phytochemical traits (the abundances of phenylpropanoid secondary metabolites in leaves), leaf morphology and growth‐related characters.

Recent results from genome‐wide association studies (GWAS) in trees, humans and other species suggest that the architecture of adaptive traits is often polygenic, that is, they are not determined by a few genes of large effect, but rather by many loci with small effect (Pritchard *et al*., 2010; Rockman, 2012; Evans *et al*., 2014; Hall *et al*., 2016; Pasaniuc & Price, 2017). Our results highlight the importance of both finite, detectable (‘sparse’) genetic loci and highly polygenic heritability of quantitative traits. Knowing these contributions is important for any in‐depth molecular genetic or genomic study aimed at dissecting complex, functionally important traits. For those traits for which ‘finite’ architectures were detected, we identified candidate genes located within associated genomic regions and we discuss potential molecular mechanisms underlying trait variation.

## Materials and Methods

### Plant materials

We analyzed seedlings of *P. alba* L., *P. tremula* L., and their hybrids, also known as *P. × canescens (Aiton) Sm*. All seeds were collected from open‐pollinated mother trees in the Parco Lombardo della Valle del Ticino in the north of Italy (Lexer *et al*., 2010; Lindtke *et al*., 2014). Seeds were sampled in 2010, 2011 and 2014 and germinated broadly following Lindtke *et al*. (2014). At *c*. 2 months after germination, we moved seedlings to larger pots and arranged them in a common garden using a block design with randomized positions within blocks. We grew *c*. 500 seedlings from 39 families in two locations: at the Botanical Garden of the University of Fribourg, Switzerland, and at the University of Salerno, Italy. Detailed information about the number of individuals per family and common garden can be found in Supporting Information Table S1.

### Genetic data

We conducted a restriction site‐associated DNA sequencing (RAD‐seq) experiment as follows. First, we extracted DNA from silica‐dried leaf material of 472 individuals using the Qiagen DNeasy Plant Mini Kit (Valencia, CA, USA) and standardized concentrations to 20 ng μl^−1^. Second, we submitted all samples to Floragenex (Eugene, OR, USA), where five libraries with 95 individuals each were prepared according to their standard commercial procedure, very similar to the original RAD‐seq protocol (Baird *et al*., 2008). Specifically, genomic DNA was digested with the restriction enzyme *Pst*I, chosen according to previous RAD‐seq studies of these species and their hybrids (Stölting *et al*., 2013; Christe *et al*., 2016), and the libraries were sequenced single‐ended on one lane of an Illumina HiSeq2500 instrument each (SRA accession number PRJNA528699). Third, we processed RAD‐seq data using state‐of‐the‐art tools, including mapping to the *P. trichocarpa* reference genome with bowtie2 2.2.4 (Langmead & Salzberg, 2012) and variant calling with gatk 3.4.46 (DePristo *et al*., 2011) following best practice (Methods S1 – scripts for these analyses and those described below are available at https://bitbucket.org/LuisaB/gwas_analyses_populus/src/master/). Fourth, we applied strict filters so as to retain only reliable sites: we removed all sites with more than segregating alleles or with an average depth above the 95% quantile to exclude potentially paralogous loci. To avoid single nucleotide variants (SNVs) originating from misalignments, we further removed indels and variant sites within 5 bp of all indels that we could identify confidently using gatk on the full data.

### Inference of genome‐wide ancestry

We estimated genome‐wide ancestry (*q*) using entropy (Gompert *et al*., 2014) directly from genotype likelihoods after removing SNVs with either minor allele frequency < 0.05 or > 50% missing data, and after correcting genotype likelihoods for biases associated with RAD‐seq (Methods S2). We further calculated *F*_ST_ between parental species using Hudson's estimator (Hudson *et al*., 1992) on the parental allele frequencies inferred with entropy.

### Inference of local ancestry

Despite substantial genetic differentiation between the parental species, they share alleles and genotypes at many loci, so that LD in our study population decays rapidly with physical distance. Thus, we used local ancestry for trait mapping, which provided greater LD along chromosomes (see below). LD is required for mapping because causal allelic variants are unlikely to be directly observed in reduced representation studies. We estimated local ancestry using raspberry (Wegmann *et al*., 2011), which implements a hidden Markov model to explain haplotypes of hybrid individuals as a mosaic of reference haplotypes provided for each species.

We obtained reference haplotypes by phasing previously characterized pure *P. alba* and *P. tremula* individuals (51 each) from the Italian, Austrian and Hungarian hybrid zones (Christe *et al*., 2016) using fastphase (Scheet & Stephens, 2006), building input files with fcgene (Roshyara & Scholz, 2014). For use in raspberry, individuals in the reference panels were not allowed to have missing data. The genotype calling step in our common garden individuals was therefore restricted to the 45 193 SNVs covered in all parental individuals (Christe *et al*., 2016). We further masked all genotype calls based on < 5 reads.

To infer local ancestries with raspberry we used the mutation rates previously estimated for *P. alba* and *P. tremula* (Christe *et al*., 2016). As a prior on the switching probabilities we further used 5 cM Mb^–1^ as the default recombination rate, as estimated for *P. trichocarpa* (Tuskan *et al*., 2006), and sample‐specific genome‐wide ancestry *q* estimated using admixture (Alexander *et al*., 2009) on all 472 individuals jointly. The remaining parameter settings, initial optimization runs, and incorporation of RAD‐seq genotyping error rates are described in detail in Methods S2.

For mapping, we then used the expected ancestry genotype calculated from the posterior probabilities obtained with raspberry. We verified the presence of LD by calculating the pairwise squared correlation between point estimates of local ancestries and visualized the results using the package ldheatmap (Shin *et al*., 2006) in R (R Core Team, 2016).

### Phenotypic data

We used 46 phenotypes, classified into phytochemical, morphological and growth traits (Tables 1, S2). For phytochemical traits, we focused on secondary metabolites in leaves from three different branches of the phenylpropanoid pathway: chlorogenic acids, salicinoids and flavonoids (see Methods S3 for more rationale on trait choice). These secondary metabolites were previously quantified for a subset of 133 samples using ultra‐high‐pressure LC quadrupole‐time‐of‐flight MS (Caseys *et al*., 2012, 2015) and we completed these measurements here for all 266 samples germinated in 2011.

**Table 1:** List of phenotypic traits analyzed in this study

Category	Trait	Abbreviation	*n* a	Covariates^b^	Binary^c^
Phytochemical, chlorogenic acid	3‐Caffeoyl quinic acid	C1	266	q, cg	no
	3‐Coumaroyl quinic acid	C2	266	q, cg	No
	5‐Caffeoyl quinic acid	C3	266	q, cg	No
	3‐Feruloyl quinic acid	C4	266	q, cg	No
	1‐Caffeoyl quinic acid	C5	266	q, cg	No
	5‐Coumaroyl quinic acid	C6	266	q, cg	No
	Coumaroyl quinic acid isomer	C6b	133	q	No
	(1,5) Dicaffeoyl quinic acid	C7	266	q, cg	No
Phytochemical, salicinoid	Salicin	C8	266	q, cg	No
	Salicortin	C9	266	q, cg	No
	Salicortin isomer 1	C9i	266	q, cg	No
	Salicortin isomer 2	C9ii	133	q	No
	Salicortin isomer 3	C9iii	266	q, cg	No
	Acetyl‐salicortin	C10	266	q, cg	No
	Acetyl‐salicortin isomer 1	C10i	266	q, cg	No
	Acetyl‐salicortin isomer 2	C10ii	266	q, cg	No
	HCH‐salicortin	C12	266	q, cg	No
	Tremuloidin	C13	266	q, cg	No
	Tremulacin	C14	266	q, cg	No
	Tremulacin isomer	C14i	266	q, cg	No
	HCH‐tremulacin	C15	266	q, cg	No
	Acetyl‐tremulacin	C16	266	q, cg	Yes
Phytochemical, flavonoid	Catechin	C17	266	q, cg	No
	Quercetin‐rutinoside‐pentose	C18	266	q, cg	No
	Quercetin‐glucuronide‐pentose	C19	266	q, cg	Yes
	Quercetin‐hexose‐pentose	C20	266	q, cg	No
	Kaempferol‐rutinoside‐pentose	C21	266	q, cg	Yes
	Isorhamnetin‐rutinoside‐pentose	C22	266	q, cg	Yes
	Quercetin‐3‐O‐rutinoside	C23	266	q, cg	Yes
	Quercetin‐3‐O‐glucuronide	C24	266	q, cg	No
	Quercetin‐3‐O‐glucoside	C25	266	q, cg	No
	Kaempferol‐3‐O‐rutinoside	C26	266	q, cg	Yes
	Isorhamnetin‐3‐O‐rutinoside	C27	266	q, cg	Yes
	Quercetin‐3‐O‐arabinopyranoside	C28	266	q, cg	Yes
	Kaempferol‐glycuronide	C29	266	q, cg	Yes
	Quercetin‐rhamnoside	C30	266	q, cg	No
	Isorhamnetin‐glycoside	C31	266	q, cg	No
	Isorhamnetin‐glycuronide	C32	266	q, cg	Yes
	Isorhamnetin‐acetyl‐hexose	C33	266	q, cg	Yes
	Isorhamnetin‐rhamnoside	C34	266	q, cg	Yes
Morphological	Leaf area	LFAREA	445	q, cg, y	–
	Leaf shape	LFSHAP	445	q, cg, y	–
Growth	Height, first year	HEIGHT1	321	q, y	–
	Height, second year	HEIGHT2	258	q, cg	–
	Diameter, first year	DIAM1	323	q, y	–
	Diameter, second year	DIAM2	258	q, cg	–

a Number of individuals with trait data.

b Covariates included: *q*, genome‐wide ancestry; cg, common garden location; *y*, planting year; PIP, for posterior inclusion probability.

c Whether the presence or absence of the chemical compound was also mapped as a binary trait in gemma.

For morphological traits, we measured the leaf area (LFAREA) and leaf shape (LFSHAP), known to be strongly divergent between *P. alba* and *P. tremula* (Lexer *et al*., 2009). To account for within‐individual variability, we followed Lindtke *et al*.(2013) and measured four leaves per plant using a ruler with a precision of 1 mm, averaged the lengths and widths for each seedling, and calculated LFAREA from these. LFSHAP was calculated by dividing the average leaf length by the average leaf width (Lindtke *et al*., 2013).

For growth traits we included measures of height and diameter of the seedlings at 1 and 2 yr after planting (HEIGHT1, HEIGHT2, DIAM1 and DIAM2). Height was quantified with a tape measure from the soil to the top of the main stem with a precision of 1 cm, whereas diameter was assessed with calipers with a precision of 1 mm at 10 cm above the soil. HEIGHT1 and DIAM1 were available for seedlings planted in 2010, seedlings planted in 2011 in Fribourg (not in Salerno) and seedlings planted in 2014. HEIGHT2 and DIAM2 were available only for seedlings planted in 2011, in both Fribourg and Salerno.

To examine how phenotypic variation relates to *q*, we quantified the proportion of phenotypic variance explained by this variable using linear regressions for each trait We then bootstrapped the data 1000 times to obtain confidence intervals.

### Admixture mapping

To carry out GWAS by admixture mapping, we used the Bayesian sparse linear mixed model (BSLMM; Zhou & Stephens, 2012) in gemma v.0.94.1 (Zhou *et al*., 2013), because it implements a polygenic approach, in which the effects of multiple loci on the phenotype are evaluated simultaneously. This provides a more complete view of genomic architecture than simpler linear models and avoids large numbers of significance tests. gemma provides estimates for a set of parameters describing the amount of phenotypic variance explained either by loci with clearly detectable effects along the chromosomes (‘sparse effects’) or by the infinitesimal effects of all markers (‘random effects’ estimated from the kinship matrix). This set of parameters includes the proportion of phenotypic variance explained by the sparse effects and random effects (PVE), the proportion of PVE explained by the sparse effects only (PGE) and the putative number of sparse effect loci involved in determining the phenotype (*n_gamma*). The product of PVE and PGE gives the proportion of total phenotypic variance explained by sparse effects, which is commonly referred to as narrow‐sense heritability *h* ^2^.

gemma also estimates the probability of each locus having a detectable sparse effect on the phenotype (the posterior inclusion probability, PIP). Neighboring SNVs in a genomic region are expected to have some redundancy and exchangeability as predictors of phenotype, and therefore to have lower individual PIPs than if a SNV tagged variation in the phenotype. To appropriately aggregate information from neighboring SNVs regarding the cumulative evidence for sparse effects in an interval, we summed PIPs in nonoverlapping windows of 0.5 Mb, as we found windows of 1 or 2 Mb resulted in similar patterns but made the identification of candidate genes harder (Fig. S1). We selected windows with a PIP ≥ 0.4 for further analysis of candidate genes, which is a higher threshold compared with other studies (Gompert *et al*., 2013; Comeault *et al*., 2014; Chaves *et al*., 2016).

To account for nonindependence among samples, and to attribute phenotypic variation to overall genetic composition of individuals (the highly polygenic component of heritability), gemma estimates a kinship matrix from the genetic data and includes it as covariate in the mixed model. As we used local ancestries as genetic input, this kinship matrix is effectively a genomic similarity matrix and captures differences in ancestry across individuals and families (Fig. S2). Before running gemma, we further regressed out *q*, the planting year and the common garden location from the phenotypes using a linear model to account for their potentially confounding effects.

For each trait, we ran 10 independent Markov chains of 12 million iterations and discarded the first two million as burn‐in. To evaluate the robustness of our conclusions, we also ran gemma including the covariates in the input file (*‐notsnp* option), rather than regressing them out. For the 12 phytochemical compounds with zero abundance in > 10% of individuals, we further coded trait values as presence (1) and absence (0) and used binomial logistic regression to obtain residuals used as phenotypic information. Additional information on gemma models and parameter settings can be found in Methods S4.

### Analysis of traits with accessible, sparse genomic architecture

We selected a core set of traits with evidence for sparse, finite architectures for further analysis. These traits had an estimated *h* ^2^ ≥ 0.01 and *n_gamma *>* *0 with at least 95% posterior probability. For these traits we then selected windows with PIP ≥ 0.4 (also for models of binary traits) and retrieved genes annotated in them in the *P. trichocarpa* reference genome (Ptrichocarpa_210_v3.0; Tuskan *et al*., 2006) and in *Arabidopsis thaliana* (The Arabidopsis Information Resource; Berardini *et al*., 2015) to identify orthologous genes. We then examined the list of genes for candidates putatively involved in the control and modulation of the phenotypes analyzed in this study.

## Results

### Genome‐wide ancestry

**Figure 1:**
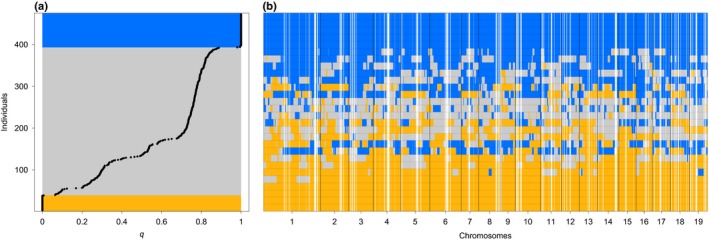
Genomic composition of genome‐wide association study (GWAS) panel. (a) Genome‐wide ancestry *q* for each common garden seedling, as estimated by entropy. Orange and blue rectangles highlight *Populus tremula* individuals (*q *<* *0.05) and *Populus alba* individuals (*q* >* *0.95), respectively, and the gray rectangle indicates hybrids. Ninety‐five per cent confidence intervals are too small to be depicted. (b) Local ancestries along the chromosomes of 28 exemplary individuals (each row is an individual), representing the range of variation of *q*. Confidence in ancestry estimates is shown by shades from white (unknown ancestry) to blue (*P. alba* ancestry), orange (*P. tremula* ancestry) or gray (heterospecific ancestry). See Supporting Information Fig. S3 for the results of all individuals.

After filtering, we kept 127 322 SNVs to infer genome‐wide ancestry (*q*) across individuals. We found considerable variation in the genomic composition of the seedlings, spanning the full range between the parental species (0 and 1; Fig. 1a). The average *F*_ST_ between the species was 0.3922, which is very similar to previous estimates based on a range of different molecular data (Lexer *et al*., 2007; Stölting *et al*., 2013; Christe *et al*., 2016). Despite this elevated differentiation, most alleles were shared between species, with only 11.6% showing allele frequency difference > 0.95.

### Local ancestry inference

We estimated local ancestry using raspberry (Wegmann *et al*., 2011) based on 32 413 SNVs passing filters and under a model with five generations since admixture, ancestral recombination rates of 500, and a miscopying rate of 0.06, which had the highest likelihood. Local ancestry analysis revealed a genomic mosaic of homospecific ancestry segments derived from *P. alba* and *P. tremula* and segments with heterospecific ancestry (Fig. 1b). As expected from genome‐wide ancestries, we observed more *alba*‐like than *tremula*‐like hybrids in our sample set (Fig. S3). Chromosomes in individuals frequently switched between homospecific segments of the two parental species without passing through a transitory region of heterospecific ancestry (Fig. S3), consistent with the well‐known challenge of correctly recovering all heterozygous genotypes in RAD‐seq experiments (Davey *et al*., 2013; Bresadola *et al*., 2019).

### Admixture LD

**Figure 2:**
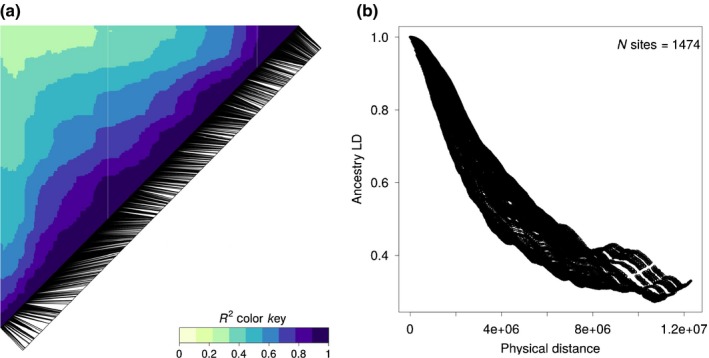
Admixture linkage disequilibrium (LD) on chromosome 9 calculated as pairwise squared correlation between point estimates of local ancestries (*R* ^2^) in an admixed seedling population of *Populus alba* and *Populus tremula*. (a) Black lines indicate the positions of analyzed loci along the chromosome, and darker blue shades represent stronger LD. (b) LD decay as a function of physical distance along the chromosome. *N* sites indicate the number of loci analyzed on this chromosome. Results for the remaining chromosomes were very similar (Supporting Information Figs S4, S5).

Successful mapping in any association study depends on the extent of LD between sites (Remington *et al*., 2001; Stracke *et al*., 2007). LD in our data displayed spatial decay patterns along chromosomes suitable for phenotype mapping: adjacent loci showed strong LD of ancestry state, which decayed gradually with physical distance (Figs 2, S4, S5).

### Phenotypic differentiation

**Figure 3:**
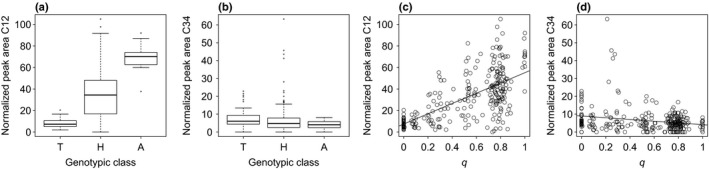
(a, b) Degrees of differentiation between *Populus alba*,* Populus tremula* and their hybrids for two phytochemical traits (C12 and C34, respectively). T, *P. tremula* (*q *<* *0.05); H, hybrid seedlings with 0.05 ≤ *q *≤* *0.95; A, *P. alba* (*q *>* *0.95). Boxes represent the first and third quartiles, whiskers extend to the lowest and highest data points within 1.5 × interquartile range (IQR) from the first and third quartiles, respectively. (c, d) Relationship between genome‐wide ancestry (*q*,* x* axis) and the two phytochemical traits. *P. tremula*‐like individuals are on the left, where *q *<* *0.05, and *P. alba*‐like individuals are on the right, where *q *>* *0.95. Hybrid seedlings exhibit intermediate values of *q*. Linear regression lines are shown as visual guides only and are not intended to suggest that a linear regression function represents the best fit to the data.

**Figure 4:**
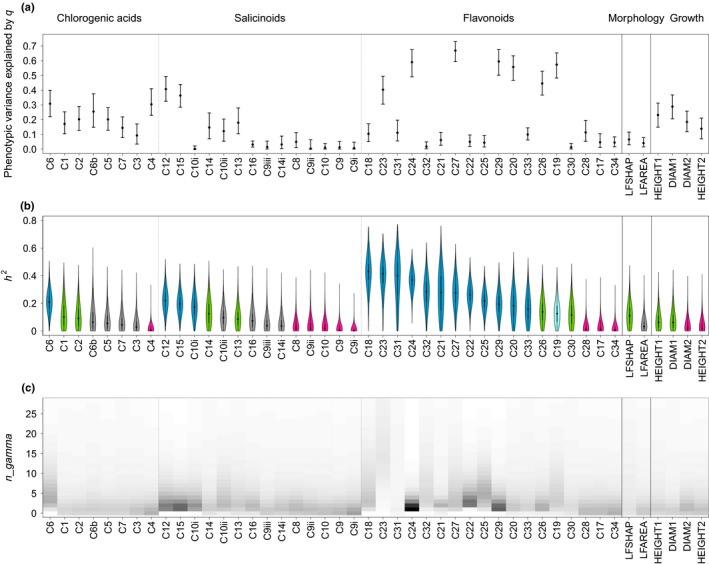
Results on genomic architecture for all traits analyzed in an admixed seedling population of *Populus alba* and *Populus tremula*, grouped based on functional similarities among traits (phytochemistry including chlorogenic acids, salicinoids, and flavonoids; morphology; growth) and ordered according to the decreasing median of narrow‐sense heritability *h* ^*2*^. (a) Proportion of phenotypic variance explained by genome‐wide ancestry *q*. Bars indicate 95% confidence intervals. (b) Violin plots showing the posterior distributions of *h* ^*2*^ (narrow‐sense heritability) of traits assigned to the first class of genomic architecture (blue), the second class (green), the third class (pink), and of traits which could not be assigned to any class (gray). C19 is shown in light blue, as it only barely missed our threshold on *h* ^2^ to be included in the first class and showed a sharp posterior inclusion probabilities peak (see the Results section and Table 2). (c) Heat map of the values of *n_gamma*, the putative number of sparse effect loci. Darker shades indicate a higher number of occurrences for the corresponding value of *n_gamma* in the posterior distribution.

Traits in our GWAS showed variable degrees of differentiation between *P. alba* and *P. tremula*. Two example traits are shown in Fig. 3: C12, which showed strong divergence between *P. alba* and *P. tremula* and intermediate values in hybrids, and C34, which exhibited similar abundance in all genotypic classes (for box plots summarizing patterns of variation for all traits, see Figs S6, S7). For some traits, we detected transgressive phenotypes in hybrids (e.g. C34; Fig. 3b,d). The proportion of phenotypic variance explained by genome‐wide ancestry (*q*) followed a heterogeneous pattern (Fig. 4a), broadly mirroring patterns of intra‐ and interspecific variability for the studied traits (Fig. S7).

### Admixture mapping

Parameter estimates obtained with the BSLMM implemented in gemma revealed a continuum of genomic architectures (Figs 4b, S8; Table S3). For further analysis, we grouped them into three main classes (Table 2):

1. The first class included traits with strong evidence for heritability and with both the genomic background and loci with measurable effect contributing to the phenotypic variation. This class of loci with strong evidence for a genetic role in explaining phenotypes included 16 phytochemical traits with *h* ^2^ ≥ 0.01 with at least 95% probability (Table 2; Figs 4b, 5a). These were the phytochemical traits showing the highest values of median *h* ^2^ and highest probability of *n_gamma *>* *0 (Fig. 4b,c). An additional trait (C19) was considered part of this class, although it did not strictly satisfy the threshold on *h* ^2^ (see later).

**Figure 5:**
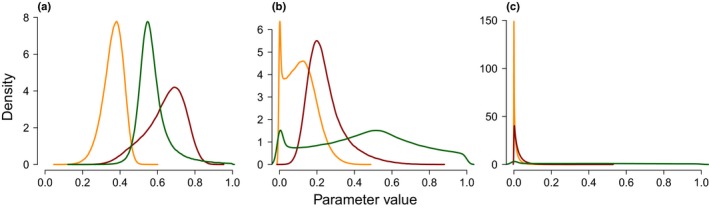
Posterior distributions of the proportion of phenotypic variance explained by the sparse effects and random effects (PVE, red), the proportion of PVE explained by the sparse effects only (PGE, green) and narrow‐sense heritability (*h* ^2^, yellow) obtained from a genome‐wide association study (GWAS) on an admixed seedling population of *Populus alba* and *Populus tremula*. Posterior distributions are shown for an exemplary trait of each class: (a) quercetin‐3‐O‐glucuronide (C24) for the first class; (b) leaf shape (LFSHAP) for the second class; and (c) 3‐feruloyl quinic acid (C4) for the third class.
2. The second class corresponds to traits for which only the genomic background appears to play a role in explaining the phenotype, while the actual contribution of individual loci with measurable effect on the phenotype is less clear. This group encompasses six phytochemical traits, the growth traits DIAM1 and HEIGHT1 and the morphological trait LFSHAP (Table 2; Figs 4b, 5b), which do not meet the heritability threshold outlined earlier, but for which PVE was ≥ 0.05 with > 97% probability.
3. The third class included traits for which both the genomic background and the variation explained by loci with measurable effect were not significantly different from zero, thus causing *h* ^2^ to approach zero. The most evident cases for this scenario were phytochemical traits C4 (Fig. 5c), C9, C9i and C34, for which *h* ^2^ < 0.01 with > 60% probability and *h* ^2^ < 0.05 with > 90% probability. The probability of *n_gamma *=* *0 was the highest for these traits. In total, nine phytochemical traits and the growth traits DIAM2 and HEIGHT2 were identified to be in this class (Tables 2, S2).

**Table 2:** Traits assigned to each class of genomic architecture, as suggested by hyperparameter posterior distributions from gemma

Trait class	Inclusion criteria	Traits^a^
First	*h* ^2^ ≥ 0.01 with > 95% probability	C6, C10i, C12, C15, C18, C20, C21, C22, C23, C24, C25, C27, C29, C31, C32, C33, C19^b^
Second	*h* ^2^ ≥ 0.01 with < 95% probability, but *PVE *≥* *0.05 with > 97% probability	C1, C2, C13, C14, C26, C30, HEIGHT1, DIAM1, LFSHAP
Third	*h* ^2^ < 0.01 with ≥ 30% probability	C4, C8, C9, C9i, C9ii, C10, C17, C28, C34, HEIGHT2, DIAM2
Not assigned	—	C3, C5, C6b, C7, C9iii, C10ii, C14i, C16, LFAREA

a C1, 3‐caffeoyl quinic acid; C2, 3‐coumaroyl quinic acid; C3, 5‐caffeoyl quinic acid; C4, 3‐feruloyl quinic acid; C5, 1‐caffeoyl quinic acid; C6, 5‐coumaroyl quinic acid; C6b, coumaroyl quinic acid isomer; C7, (1,5) dicaffeoyl quinic acid; C8, salicin; C9, salicortin; C9i, salicortin isomer 1; C9ii, salicortin isomer 2; C9iii, salicortin isomer 3; C10, acetyl‐salicortin; C10i, acetyl‐salicortin isomer 1; C10ii, acetyl‐salicortin isomer 2; C12, HCH‐salicortin; C13, tremuloidin; C14, tremulacin; C14i, tremulacin isomer; C15, HCH‐tremulacin; C16, acetyl‐tremulacin; C17, catechin; C18, quercetin‐rutinoside‐pentose; C19, quercetin‐glucuronide‐pentose; C20, quercetin‐hexose‐pentose; C21, kaempferol‐rutinoside‐pentose; C22, isorhamnetin‐rutinoside‐pentose; C23, quercetin‐3‐O‐rutinoside; C24, quercetin‐3‐O‐glucuronide; C25, quercetin‐3‐O‐glucoside; C26, kaempferol‐3‐O‐rutinoside; C27, isorhamnetin‐3‐O‐rutinoside; C28, quercetin‐3‐O‐arabinopyranoside; C29, kaempferol‐glycuronide; C30, quercetin‐rhamnoside; C31, isorhamnetin‐glycoside; C32, isorhamnetin‐glycuronide; C33, isorhamnetin‐acetyl‐hexose; C34, isorhamnetin‐rhamnoside; LFAREA, leaf area; LFSHAP, leaf shape; HEIGHT1, height, first year; HEIGHT2, height, second year; DIAM1, diameter, first year; DIAM2, diameter, second year.

b This trait does not strictly satisfy the requirements for the first class, but was included because it showed a strong signal for heritability (*h* ^2^ ≥ 0.01 with > 93% probability – Supporting Information Table S3) and a sharp posterior inclusion probability peak (see text).

For the remaining traits (eight phytochemical traits and LFAREA; Table 2), the posterior distributions of *h* ^2^ did not allow us to obtain clear insights concerning their genomic architecture. It was therefore not possible to assign them to a specific class.

### Analysis of focal traits with finite genomic architecture

We identified the genomic regions with sparse effects for the 16 traits in the first class of genomic architectures. All of these were phytochemical traits, that is, secondary metabolite compounds: one chlorogenic acid (C6, *5‐coumaroyl quinic acid*), three salicinoids (C10i, *acetyl‐salicortin isomer 1*; C12, *HCH‐salicortin*; C15, *HCH‐tremulacin*) and 12 flavonoids (C18, *quercetin‐rutinoside‐pentose*; C20, *quercetin‐hexose‐pentose*; C21, *kaempferol‐rutinoside‐pentose*; C22, *isorhamnetin‐rutinoside‐pentose*; C23, *quercetin‐3‐O‐rutinoside*; C24, *quercetin‐3‐O‐glucuronide*; C25, *quercetin‐3‐O‐glucoside*; C27, *isorhamnetin‐3‐O‐rutinoside*; C29, *kaempferol‐glycuronide*; C31, *isorhamnetin‐glycoside*; C32, *isorhamnetin‐glycuronide*; C33, *isorhamnetin‐acetyl‐hexose*). As mentioned earlier, we investigated an additional trait as part of this set (C19, *quercetin‐glucuronide‐pentose*), as it exhibited a genomic window with PIP ≥ 0.4 and only barely missed our threshold on *h* ^2^ with a posterior probability > 93%.

Eleven traits with finite effects were associated with a single genomic window with PIP ≥ 0.4 per trait, whereas traits C18, C22, C23, C31, C32 exhibited two or three windows above this threshold. C33 had no genomic window with PIP ≥ 0.4, despite satisfying the requirements regarding *h* ^2^ and *n_gamma*. As the phytochemical compounds underlying traits C19, C29 and C32 were not produced by > 10% of individuals, we also conducted mapping on the presence or absence of these compounds (binary analysis; Notes S1). This analysis also revealed identifiable sparse effects, but only for C29 did the signal reside in the same genomic window as in the quantitative analysis. For traits C19 and C32, in contrast, the identified windows did not overlap, suggesting that different variants are responsible for downregulating or inhibiting the pathway leading to these compounds.

**Figure 6:**
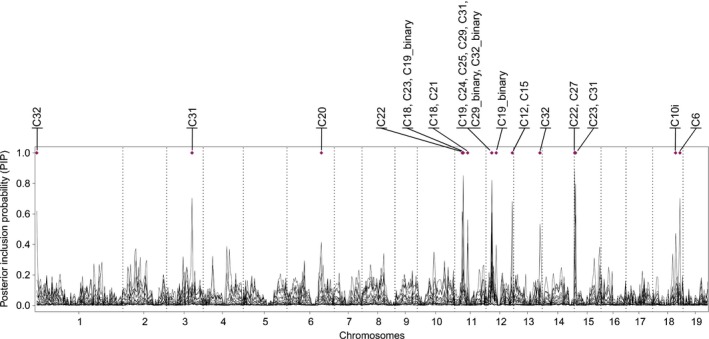
Values of posterior inclusion probabilities (PIPs) per 0.5 Mb windows for all selected traits in an admixed seedling population of *Populus alba* and *Populus tremula*. Windows with PIP ≥ 0.4 are marked with a violet diamond and the traits where this threshold is exceeded are indicated.

Windows of special interest were located on chromosomes 1, 3, 6, 11, 12, 13, 15 and 18 (Fig. 6; Notes S2; Table S4). Several windows showed PIP peaks for more than one trait: this was the case for two windows on each of the chromosomes 11, 12 and 15. Particularly interesting is the window between 3 and 3.5 Mb on chromosome 12, which appears to be involved in explaining six different phytochemical traits, all belonging to the flavonoid branch of the phenylpropanoid pathway. The results obtained with alternative modeling options in gemma (Methods S3) corroborated those obtained with our primary approach (Notes S1).

### Candidate genes in windows with high PIP

Within the windows of interest (PIP ≥ 0.4), we identified several candidate genes potentially responsible for the significant associations between specific genomic regions and phenotypic traits. Most conspicuously, these windows contained genes encoding *MYB‐type transcription factors* known to function in combination in plants (*P. trichocarpa* gene models Potri.001G005100, Potri.013G149100, and Potri.013G149200; Höll *et al*., 2013; Liu *et al*., 2014); several *UDP‐glycosyl transferases*, that is, proteins able to transfer sugar moieties with important roles in phenylpropanoid compound biosynthesis (e.g. Potri.011G060300 and Potri.011G061000; Aksamit‐Stachurska *et al*., 2008; Babst *et al*., 2014; Caseys *et al*., 2015); a *glycosyl hydrolase* involved in the biosynthetic flow by reducing the complexity of sugars of compounds (Potri.015G010100); COMT1, a *methyl transferase* that transforms quercetins into isorhamnetins (Potri.015G003100); and CHS, a *chalcone synthase* (Potri.012G138800). More information on candidate genes found in genomic windows with high PIP is provided in Notes S2 and Table S4, and the most plausible candidate genes are discussed in the following.

## Discussion

Early genetic mapping studies have often pointed to potentially simple, sparse genetic architectures of phenotypic traits in wild and domesticated species (reviewed by, e.g., Coyne & Orr, 2004), but we now know that adaptive traits are often polygenic (e.g. Pritchard *et al*., 2010; Rockman, 2012; Evans *et al*., 2014; Pasaniuc & Price, 2017) or possibly even ‘omnigenic’ (Boyle *et al*., 2017). Given sufficient power and suitable analytical tools, a reasonable expectation for trait architectures uncovered by quantitative trait locus (QTL) mapping or GWAS studies may thus be no or only very subtle genetic effects, unless multiple small‐effect mutations or pleiotropic effects accumulate in the same hotspot of phenotypic evolution (Martin & Orgogozo, 2013), the traits are very tightly coupled with (or the direct products of) the underlying biochemical pathways (e.g. Boeckler *et al*., 2011), or the focus is on traits segregating between highly divergent populations, for which architectures may differ from classical within‐species expectations (Rieseberg & Buerkle, 2002; Lexer *et al*., 2005). In this study, we investigated the genomic architecture of phenotypic trait differences between two ecologically divergent forest tree species (*P. alba* and *P. tremula*) by applying GWAS to an admixed population.

### Variation available for genetic mapping in admixed populations

Admixture mapping studies require sufficient genetic and phenotypic variation to uncover genetic associations and trait architectures (Briscoe *et al*., 1994; reviewed by Buerkle & Lexer, 2008). Our analysis of genomic variation confirmed that these two poplar species, besides their ecological divergence, are also strongly divergent genetically at many loci (mean *F*_ST_ = 0.3922), in line with previous estimates (Lexer *et al*., 2010; Stölting *et al*., 2013; Christe *et al*., 2017). This resulted in favorable conditions for estimating local ancestry (Christe *et al*., 2016) and thus for mapping. Divergence at the genetic level was reflected by pronounced differentiation at the phenotypic level for a range of characters (Figs 3, S6, S7), with several traits showing strong differences between the parental species, especially in the case of phytochemical traits. Hybrids showed intermediate or parental‐like values for most traits. Nevertheless, many recombinant hybrids showed phenotypic values falling outside the range of variation of the parental species (Figs 3b,d, S6, S7) and are thus examples of transgressive segregation (Rieseberg *et al*., 2003; Dittrich‐Reed & Fitzpatrick, 2013).

### Traits with high, medium or low heritability

Our GWAS identified genomic architectures that fall roughly into three main classes. The first class consisted of traits with evidence for relatively high heritability *h* ^*2*^ and for which a finite set of genomic regions contribute to the phenotype. All these were phytochemical traits, a finding consistent with the notion that finite genetic effects are more easily detected for traits tightly coupled with the underlying pathways.

The second class included traits for which phenotypic variation is explained by genetic effects as detected by PVE captured by our kinship (= genomic similarity) matrix, but no localized association was detected (PGE *c*. zero). One likely reason why the genomic similarity matrix explains phenotypic variation is that the trait is heritable but highly polygenic, as was previously reported for growth‐related traits (Wood *et al*., 2014; Tsai *et al*., 2015), and also in the case of *Populus* species (Du *et al*., 2016). It is also possible that sparse effects are underestimated (see later).

The third class consisted of traits for which we did not recover any evidence for heritability. Many of these are phytochemical defense traits against herbivores, which may be predominantly influenced by environmental factors (Abreu *et al*., 2011; Boeckler *et al*., 2011). However, some of these traits did show considerable phenotypic differences between the species, and this was true in our common garden setting as well. The potential lack of a heritable signal could therefore also indicate a lack of power of our admixture mapping approach. Indeed, many causal variants were probably missed by our reduced representation sequencing experiment, and were also not well tagged as a result of generally very low LD in *Populus* (a few hundred base pairs according to Ingvarsson, 2008; Marroni *et al*., 2011; but see Olson *et al*., 2010; Slavov *et al*., 2012). To mitigate this issue, however, we chose to conduct mapping on local ancestry, which exhibits long‐range LD among the early generation hybrids used here. A more likely cause for the inferred trait architectures is thus that some of these traits are highly polygenic, and a failure to detect significant heritability for such traits might be a result of a lack of power associated with admixture mapping. Residual error in RAD‐seq genotype calls may have contributed to reduced power in the present experiment, despite the use of dedicated correction procedures to mitigate genotyping error as far as possible (see earlier). Also, it is possible that effect sizes of major QTLs were slightly overestimated as a result of statistical bias, also known as the Beavis effect (Beavis, 1998). Nevertheless, our results provide indications regarding which traits (or types of traits) are more readily amenable to subsequent genetic analysis and experiments, because they exhibit more easily detectable, finite effects.

### Low heritability of growth‐related traits

Heritability estimates were conspicuously low for growth‐related traits, despite controlling for potential covariates. A lack of heritable variation for growth traits was previously observed in poplar and willow species (Orians *et al*., 2003; Du *et al*., 2016). The PVE estimates we observed are probably mainly as a result of the phenotypic variance that can be explained by the genomic ancestry similarity matrix (Zhou *et al*., 2013), as discussed earlier.

### Pleiotropic effects among phytochemical traits

Out of the 14 genomic windows exhibiting high values of PIP, six showed significant association with more than one phytochemical trait. This suggests that loci controlling different compounds are in linkage in the same window, or that the same loci are responsible for several traits, that is, that they have pleiotropic effects. One indication suggesting pleiotropic effects is that the traits associated with the same window always belong to the same branch of the phenylpropanoid pathway (five windows associated with several flavonoids and one window associated with two salicinoids). These windows could host enzymes acting upstream in a specific pathway branch, thus affecting several downstream steps and compounds (Cork & Purugganan, 2004).

### Candidate genes associated with phenylpropanoid compounds

We identified several noteworthy candidate genes in PIP peaks for flavonoid traits that code for glycosyl transferases. These enzymes act in glycosylation (i.e. conjugation to a sugar moiety), one of the most widespread modifications of plant secondary metabolites (Gachon *et al*., 2005), effectively modifying solubility, stability and reactivity of compounds (Aksamit‐Stachurska *et al*., 2008). Their association with genes within PIP peaks for flavonoid traits is consistent with their previously inferred involvement in transgressive expression of phytochemical traits in an overlapping sample of poplar hybrids (Caseys *et al*., 2015). Among other noteworthy candidate genes (Notes S2), a chalcone‐synthase (CHS) within a PIP peak for HCH‐salicinoids (two highly toxic compounds) is of particular interest: this gene, currently assigned to the flavonoid pathway, may instead be active in the largely unknown salicinoid pathway. We hypothesize that the polyketide synthase activity may act directly on benzoyl‐CoA, which has recently been put forward as a likely precursor to this entire group of compounds (Notes S2).

The genomic windows with high PIP also yielded a shortlist of candidate genes for the biosynthesis of the studied phenylpropanoid compounds and its regulation (Table S4). The flavonoid isorhamnetin‐glycuronide (C32), for example, was significantly associated with two windows hosting three transcription factors of the MYB family previously shown to regulate the phenylpropanoid pathway (Sablowski *et al*., 1994; Liu *et al*., 2015). The candidate genes MYB14 and MYB15 were found to interact in plants to stimulate the production of stilbenes, a group of phenylpropanoid compounds produced in response to biotic and abiotic stresses (Höll *et al*., 2013; Duan *et al*., 2016). MYB15 also confers improved tolerance to drought and salt stress (Ding *et al*., 2009), negatively regulates the expression of CBFs (genes expressed in response to cold conditions; Chinnusamy *et al*., 2007), and regulates defense‐induced lignification and basal immunity in *A. thaliana* (Chezem *et al*., 2017). Finally, the transcription factor MYB5, which is under positive selection in *P. tremula* (Christe *et al*., 2017), interacts physically with MYB14 and activates the promoter of enzymes related to the biosynthesis of proanthocyanidins (Liu *et al*., 2014), a major class of flavonoids responsible for color in various plant organs.

These findings are remarkable from the perspective of reproductive isolation between divergent species and, in particular, the breakdown of hybrid fitness in hybrids of *P. alba* and *P. tremula* (Christe *et al*., 2016): postzygotic reproductive barriers could originate from the disruption of coadapted gene complexes, when proper interactions between gene products cannot take place and, consequently, hybrids show a nonfunctional phenotype (Ortíz‐Barrientos *et al*., 2007; Livingstone *et al*., 2012; Lindtke & Buerkle, 2015). These interacting MYB transcription factors might represent an example of this mechanism at work: their involvement in plant defense could cause adverse effects in plants carrying incompatible genotypes, thus affecting plant survival and performance in early life stages when selection in trees tends to be strong (Petit & Hampe, 2006).

## Author contributions

CL and LB conceived the study; CL provided funding; LB, CC and SC collected genetic and phenotypic data; LB and CAB performed the analyses; CC collected information and provided valuable insights regarding candidate genes; CAB, DW and CL supervised the study; and LB, DW and CL wrote the manuscript with input and revisions from all co‐authors.

## Supporting information

Please note: Wiley Blackwell are not responsible for the content or functionality of any Supporting Information supplied by the authors. Any queries (other than missing material) should be directed to the *New Phytologist* Central Office.

**Fig. S1** Posterior inclusion probabilities in genomic windows of different size.
**Fig. S2** Kinship matrix calculated by Gemma.
**Fig. S3** Local ancestries for all analyzed seedlings.
**Fig. S4** Admixture linkage disequilibrium in all chromosomes.
**Fig. S5** Decay of admixture linkage disequilibrium along all chromosomes.
**Fig. S6** Degrees of phenotypic differentiation between *P. tremula*, hybrids and *P. alba* for all traits.
**Fig. S7** Relationship between genome‐wide ancestry and phenotype for all traits.
**Fig. S8** Posterior distributions of PVE, PGE and heritability for all phenotypic traits.
**Methods S1** RAD‐seq data processing, reference‐mapping and variant calling.
**Methods S2** Inference of local and genome‐wide ancestry.
**Methods S3** Rationale for choice of plant traits measured in this study.
**Methods S4** Admixture mapping with gemma: model choice and validation.
**Notes S1** Genomic windows highlighted by alternative modeling approaches in gemma.
**Notes S2** Additional information on candidate genes.
**Table S1** Number of seedlings per family and common garden location.
**Table S2** Phenotypic data used in this admixture mapping GWAS study.
**Table S3** Probabilities from posterior distributions of heritability, PVE, PGE and *n_gamma*.
**Table S4** Candidate genes in genomic windows with high posterior inclusion probability.
