# Supplementary material for: Admixture mapping in interspecific Populus hybrids identifies classes of genomic architectures for phytochemical, morphological and growth traits
Source: New Phytol. 2019 Jun 23;223(4):2076–89. doi: 10.1111/nph.15930 (PMC6771622; doi:10.1111/nph.15930)
Supplement: Supplementary file 1 — Fig. S1 Posterior inclusion probabilities in genomic windows of different size. Fig. S2 Kinship matrix calculated by Gemma. Fig. S3 Local ancestries for all analyzed seedlings. Fig. S4 Admixture linkage disequilibrium in all chromosomes. Fig. S5 Decay of admixture linkage disequilibrium along all chromosomes. Fig. S6 Degrees of phenotypic differentiation between P. tremula, hybrids and P. alba for all traits. Fig. S7 Relationship between genome‐wide ancestry and phenotype for all traits. Fig. S8 Posterior distributions of PVE, PGE and heritability for all phenotypic traits. Methods S1 RAD‐seq data processing, reference‐mapping and variant calling. Methods S2 Inference of local and genome‐wide ancestry. Methods S3 Rationale for choice of plant traits measured in this study. Methods S4 Admixture mapping with gemma: model choice and validation. Notes S1 Genomic windows highlighted by alternative modeling approaches in gemma. Notes S2 Additional information on candidate genes. Table S1 Number of seedlings per family and common garden location. Table S2 Phenotypic data used in this admixture mapping GWAS study. Table S3 Probabilities from posterior distributions of heritability, PVE, PGE and n_gamma. Table S4 Candidate genes in genomic windows with high posterior inclusion probability. [file NPH-223-2076-s001.pdf]

New Phytologist **Supporting Information**

Article title: Admixture mapping in interspecific *Populus* hybrids identifies classes of genomic architectures for phytochemical, morphological and growth traits
Authors: Luisa Bresadola, Céline Caseys, Stefano Castiglione, C. Alex Buerkle*, Daniel Wegmann*, Christian Lexer*

* *joint senior authors*
Article acceptance date: 06 May 2019

The following Supporting Information is available for this article:

Fig. S1 Posterior inclusion probabilities in genomic windows of different size

Fig. S2 Kinship matrix calculated by GEMMA

Fig. S3 Local ancestries for all analyzed seedlings

Fig. S4 Admixture linkage disequilibrium in all chromosomes

Fig. S5 Decay of admixture linkage disequilibrium along all chromosomes

Fig. S6 Levels of phenotypic differentiation between *P. tremula*, hybrids and *P. alba* for all traits

Fig. S7 Relationship between genome-wide ancestry and phenotype for all traits

Fig. S8 Posterior distributions of*PVE*, *PGE* and heritability for all phenotypic traits

Table S1 Number of seedlings per family and common garden location

Table S2 Phenotypic data used in this admixture mapping GWAS study

Table S3 Probabilities from posterior distributions of heritability, *PVE*, *PGE* and *n_gamma*

Table S4 Candidate genes in genomic windows with high posterior inclusion probability

Methods S1RAD-seq data processing, reference-mapping and variant calling

Methods S2 Inference of local and genome-wide ancestry

Methods S3 Rationale for choice of plant traits measured in this study

Methods S4 Admixture mapping with GEMMA: model choice and validation

Notes S1 Genomic windows highlighted by alternative modeling approaches in GEMMA

Notes S2 Additional information on candidate genes

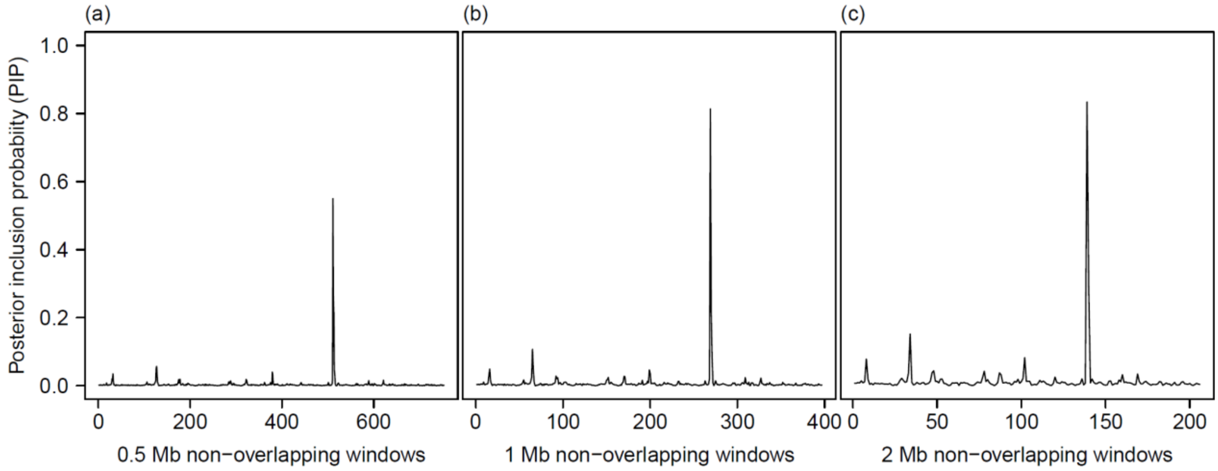

Fig. S1. Posterior Inclusion Probabilities (PIP) summed within windows of 0.5 Mb (**a**), 1 Mb (**b**) or 2 Mb (**c**). PIP patterns of C24 are shown as an example.

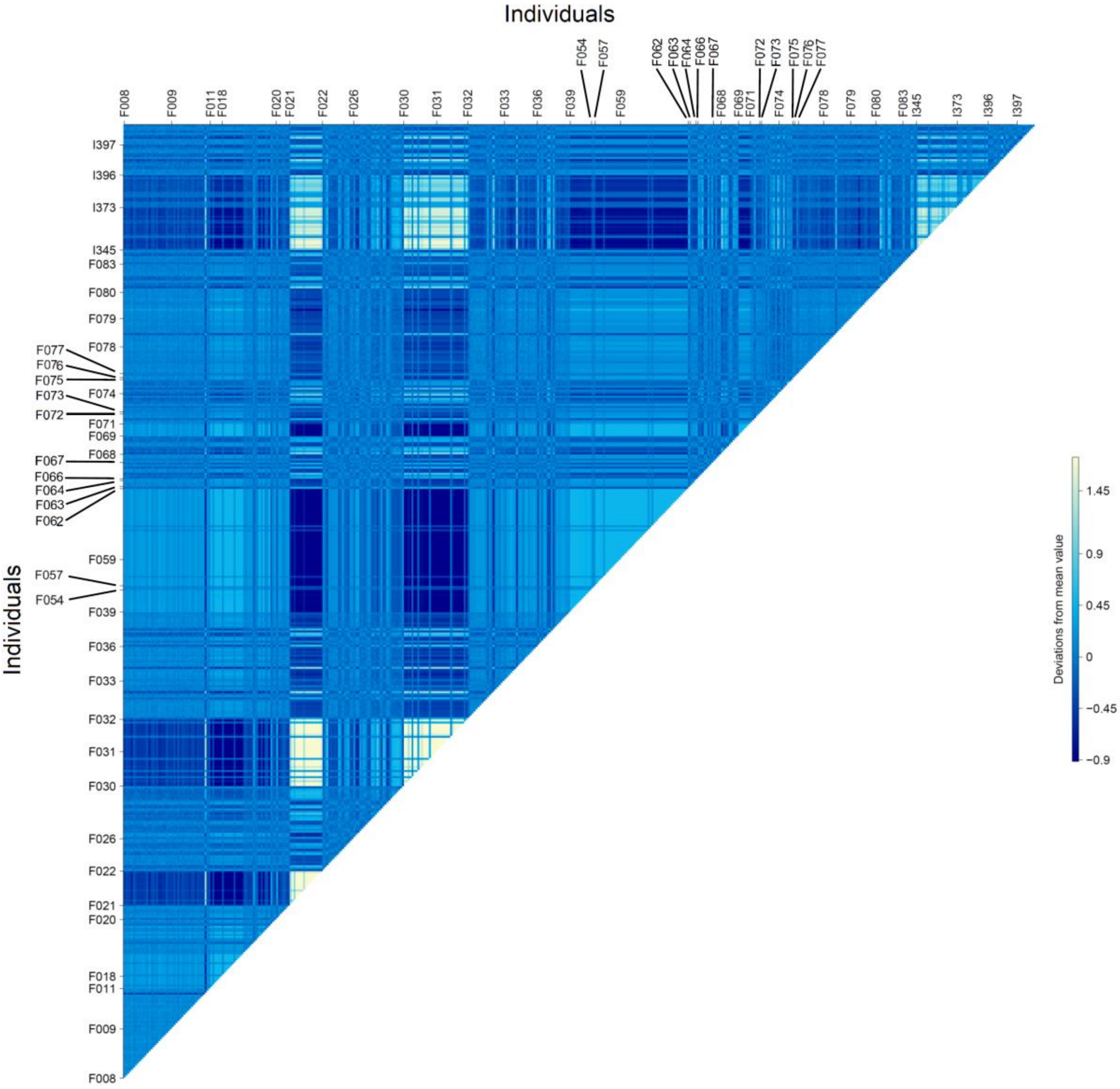

Fig. S2. Centered kinship matrix calculated by GEMMA. Each square represents the relatedness between a pair of individuals for inferred ancestry states. Color shades represent the deviation of the relatedness between a pair of individuals from the mean relatedness value. Labels indicate name of the seedling families and their location corresponds to the first individual of the family.

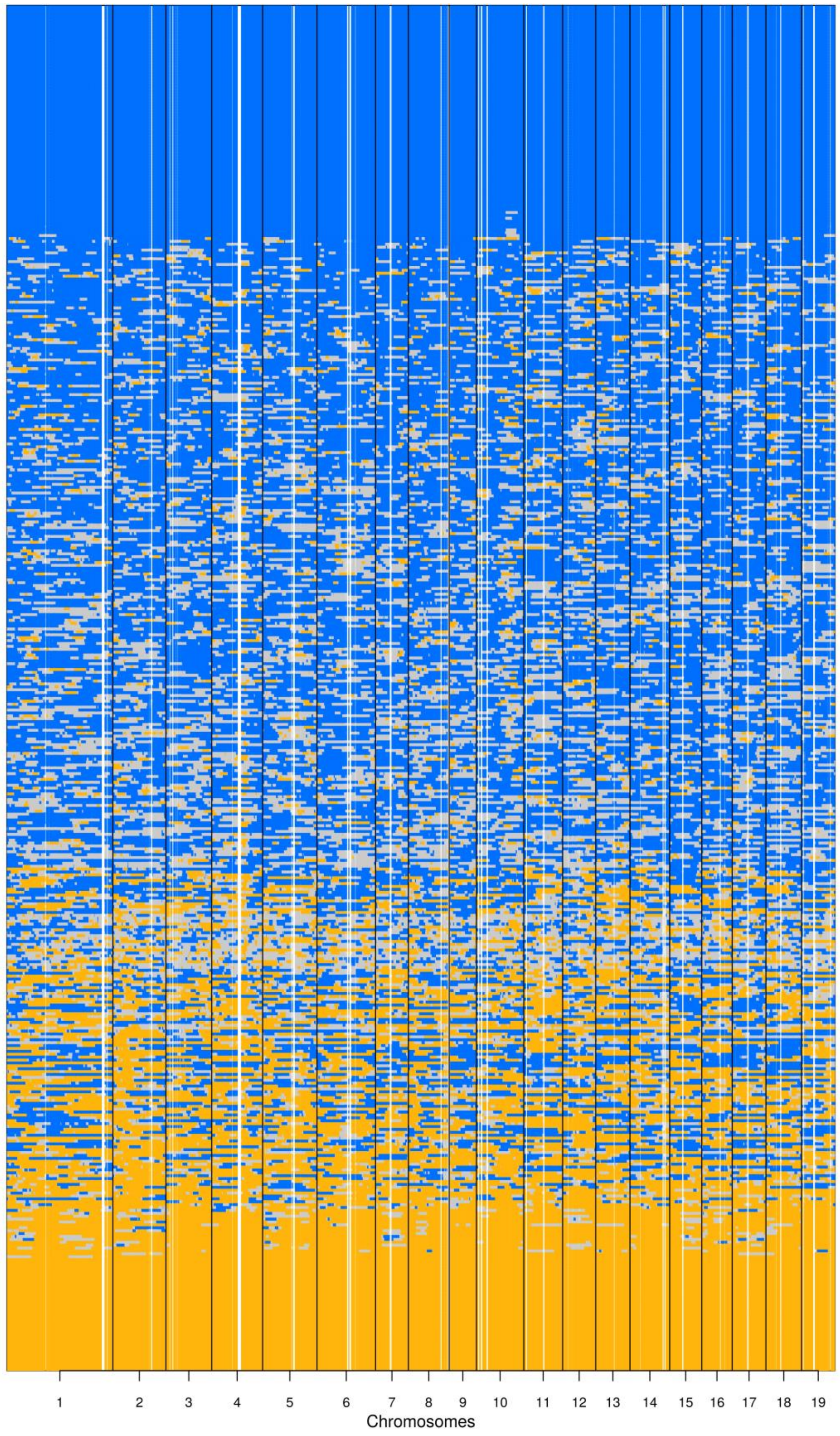

Fig. S3. Summary of local ancestries along the chromosomes (x axis) of 472 common garden seedlings, ordered along the y axis according to their genome-wide ancestry (each row is an individual). Blue represents chromosomal segments with *P. alba* ancestry, grey indicates hetero-specific ancestry, orange *P. tremula* ancestry. Darker color shades indicate more confidence in the local ancestry estimate.

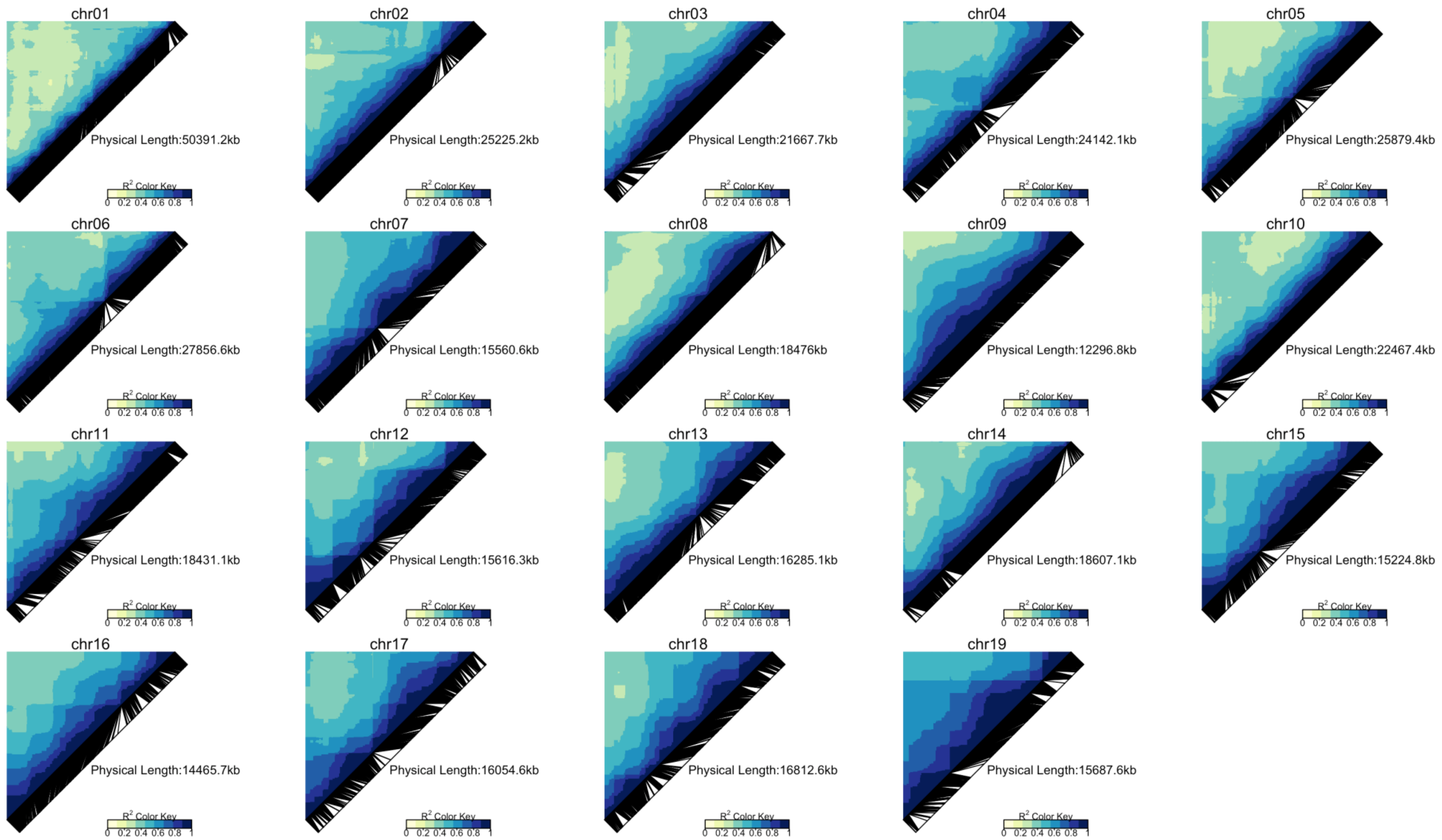

Fig. S4. Admixture linkage disequilibrium (LD) in all chromosomes, calculated as pairwise squared correlation between averaged ancestries. Black lines indicate the position of analyzed loci along the chromosome and darker blue shades represent stronger LD.

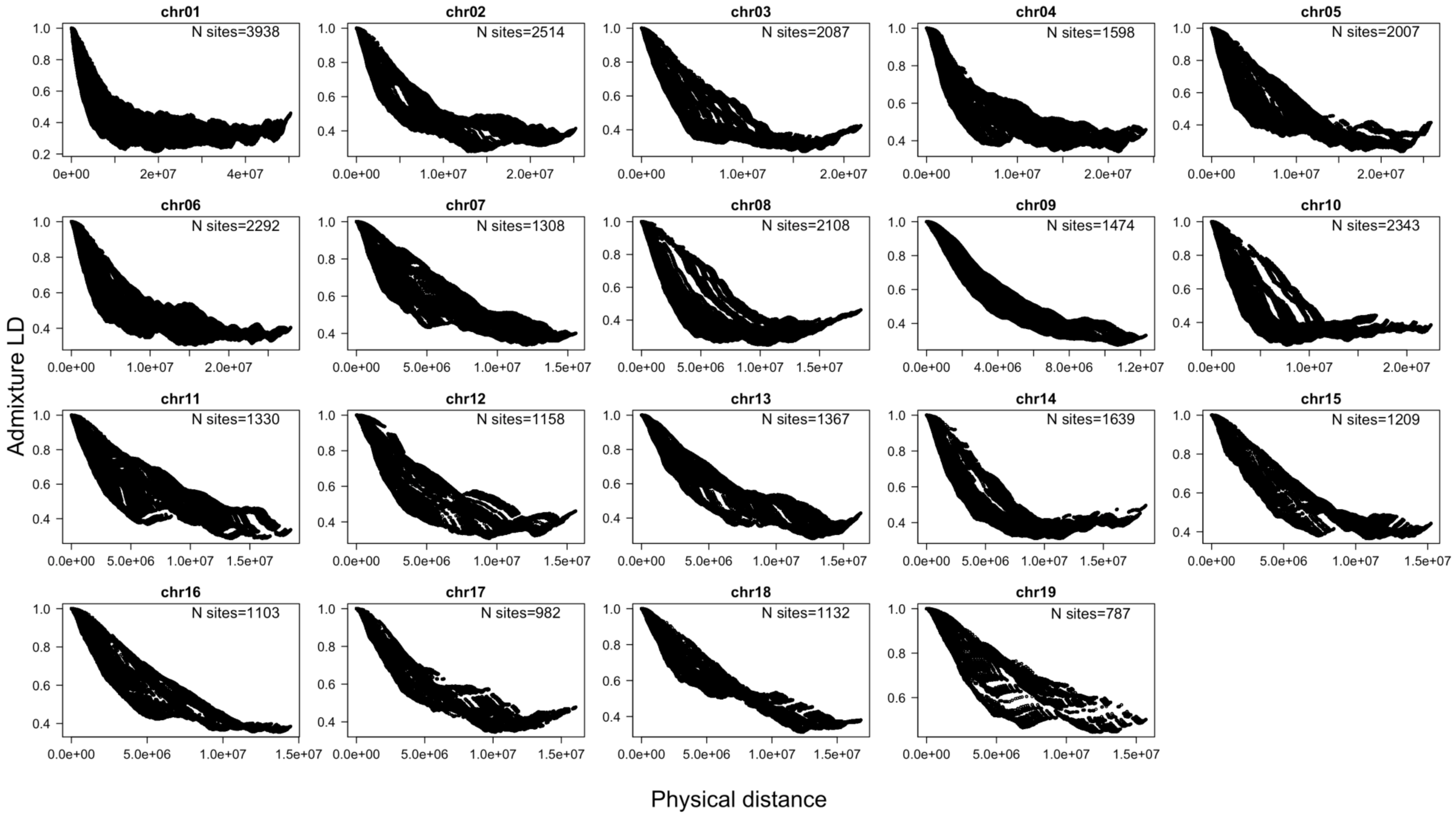

Fig. S5. Decay of admixture LD with physical distance shown for all chromosomes. *N sites* represents the number of markers available for each chromosome.

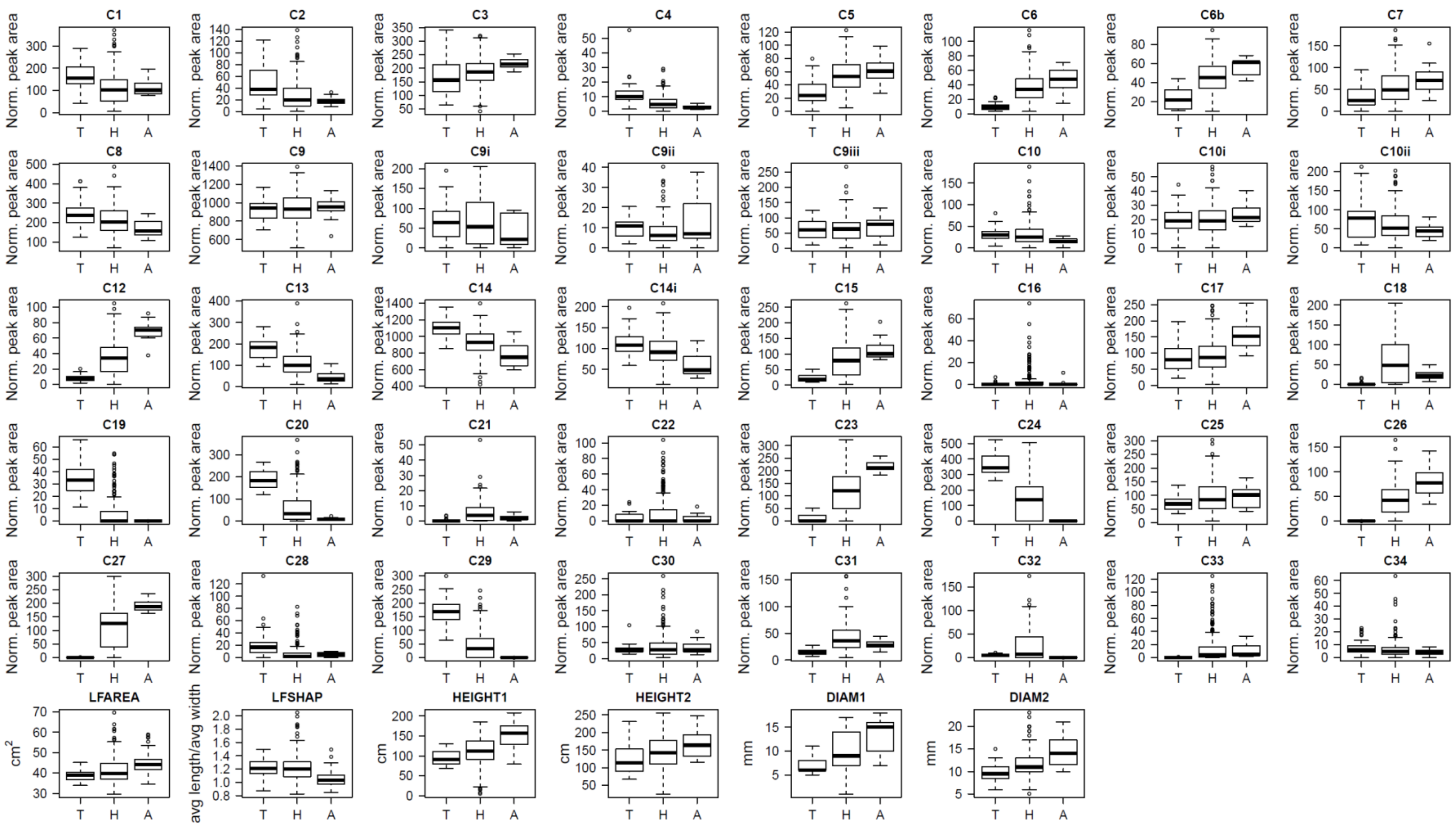

Fig. S6. Levels of differentiation between *P. tremula* (T), hybrids (H) and *P. alba* (A) for all traits analyzed in this study. Boxes represent the first and third quartiles, whiskers extend to the lowest and highest data points within 1.5 x IQR (interquartile range) from the first and third quartile, respectively.

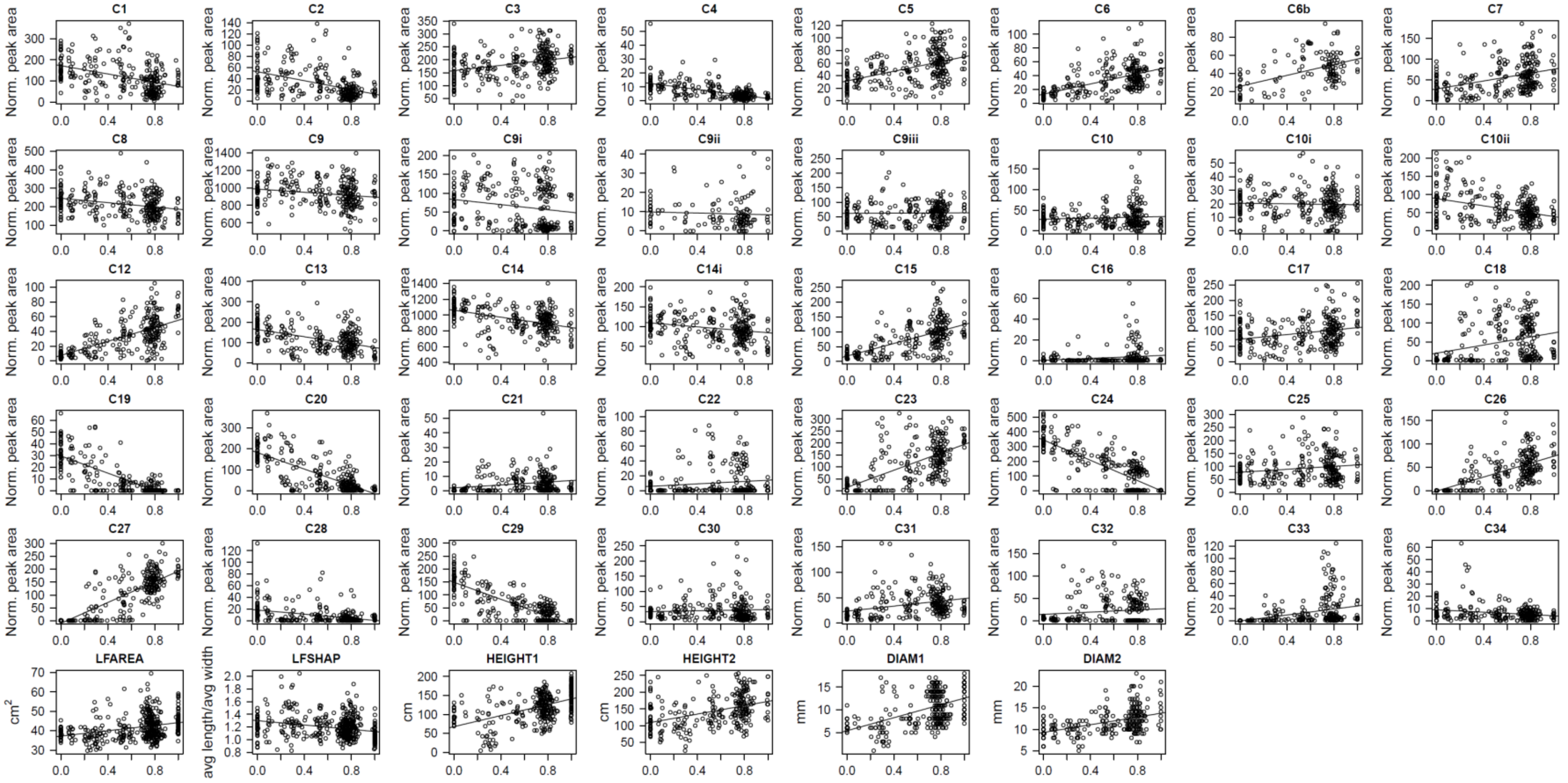

Fig. S7. Relationship between genome-wide ancestry (*q*) and the phenotypic traits. *P. tremula*-like individuals are on the left, where *q* <0.05, while *P. alba*-like individuals are on the right, where *q* >0.95. Hybrid seedlings exhibit intermediate values of *q*. Linear regression lines are shown as visual guides only and are not intended to suggest that a linear regression function represents the best fit to the data.

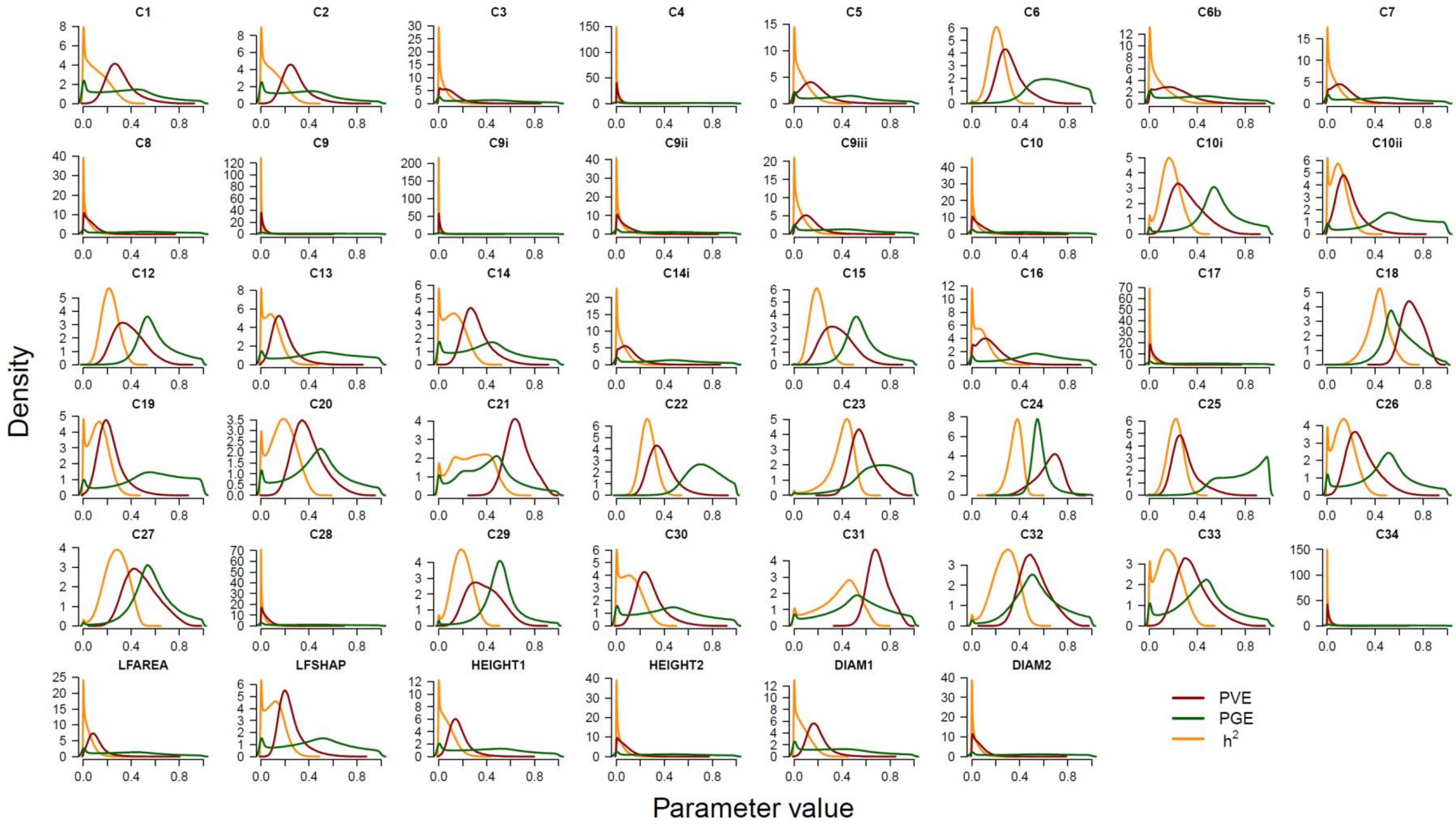

Fig. S8. Posterior distributions for *PVE*, *PGE* and heritability h^2 for all phenotypic traits.

Table S1. Overview of common garden seedling families, ordered by planting year. The number of individuals per family per common garden location is reported for each phenotypic trait analyzed in the study. *y* = planting year, *cg* = common garden location (*FR* = Fribourg and *SA* = Salerno).

Family	y	cg	Phenotypic traits					
			C1, C2, C3, C4, C5, C6, C7, C8, C9, C9i, C9iii, C10, C10i, C10ii, C12, C13, C14, C14i, C15, C16, C17, C18, C19, C20, C21, C22, C23, C24, C25, C26, C27, C28, C29, C30, C31, C32, C33, C34	C6b, C9ii	LFAREA, LFSHAP	HEIGHT1	DIAM1	HEIGHT2, DIAM2
F011	2010	FR	-	-	6	6	6	-
F018	2010	FR	-	-	28	28	28	-
F008	2011	FR	17	17	17	17	17	17
		SA	8	-	8	-	-	8
F009	2011	FR	13	13	13	13	13	13
		SA	7	-	7	-	-	7
F020	2011	FR	7	7	7	7	7	6
F021	2011	FR	7	7	7	7	7	5
		SA	10	-	10	-	-	10
F022	2011	FR	7	7	7	7	7	7
		SA	9	-	9	-	-	9
F026	2011	FR	16	16	16	16	16	16
		SA	9	-	9	-	-	9
F030	2011	FR	7	7	7	7	7	7
		SA	10	-	10	-	-	10
F031	2011	FR	7	7	7	7	7	6
		SA	9	-	6	-	-	9
F032	2011	FR	13	13	13	13	13	12
		SA	6	-	6	-	-	6

F033	2011	FR	7	7	7	7	7	7
		SA	10	-	10	-	-	10
F036	2011	FR	5	5	5	5	5	3
		SA	10	-	10	-	-	10
F039	2011	FR	7	7	7	7	7	7
		SA	4	-	4	-	-	4
I345	2011	FR	7	7	7	7	7	7
		SA	14	-	11	-	-	13
I373	2011	FR	7	7	7	7	7	7
		SA	9	-	9	-	-	9
I396	2011	FR	6	6	6	6	6	6
		SA	8	-	6	-	-	8
I397	2011	SA	10	-	8	-	-	10
F054	2014	FR	-	-	2	2	2	-
F057	2014	FR	-	-	13	13	13	-
F059	2014	FR	-	-	34	33	34	-
F062	2014	FR	-	-	1	1	1	-
F063	2014	FR	-	-	3	3	3	-
F064	2014	FR	-	-	1	1	1	-
F066	2014	FR	-	-	5	5	6	-
F067	2014	FR	-	-	4	4	4	-
F068	2014	FR	-	-	7	7	7	-
F069	2014	FR	-	-	6	6	6	-
F071	2014	FR	-	-	4	4	4	-
F072	2014	FR	-	-	1	1	1	-
F073	2014	FR	-	-	7	7	7	-
F074	2014	FR	-	-	6	6	6	-
F075	2014	FR	-	-	1	1	1	-
F076	2014	FR	-	-	2	2	2	-
F077	2014	FR	-	-	13	13	13	-

F078	2014	FR	-	-	14	14	14	-
F079	2014	FR	-	-	12	12	12	-
F080	2014	FR	-	-	13	13	13	-
F083	2014	FR	-	-	6	6	6	-
Total			266	133	445	321	323	258

Table S2. Phenotypic data used in this admixture mapping GWAS study (enclosed as a separate spreadsheet dataset).

Table S3. Probabilities from posterior distributions for the parameters heritability (h^2), *PVE*, *PGE* and *n_gamma*. Probabilities are rounded to the fourth decimal. Traits marked with a star satisfied the requirements explained in Materials and Methods and their genomic windows with Posterior Inclusion Probability (PIP) ≥0.4 were searched for candidate genes. An additional trait (C19) was added to the list of focal traits (see Results and Table 2 for details).

Traits	Probabilities						
	h^2<0.01	h^2<0.05	*PVE*<0.01	*PVE*<0.05	*PGE*<0.01	*PGE*<0.05	*n_gamma*>0
C1	0.1165	0.2993	0.0001	0.0010	0.0709	0.1237	0.9505
C2	0.1256	0.3250	0	0.0009	0.0748	0.1307	0.9481
C3	0.2955	0.6628	0.0667	0.2870	0.0881	0.1337	0.9311
C4	0.6645	0.9601	0.3662	0.8568	0.1179	0.1652	0.9024
C5	0.1806	0.4679	0.0210	0.1094	0.0690	0.1124	0.9485
C6*	0.0029	0.0097	0	0.0001	0.0016	0.0031	0.9989
C6b	0.1847	0.4350	0.0277	0.1240	0.0725	0.1175	0.9463
C7	0.2113	0.5249	0.0355	0.1746	0.0699	0.1101	0.9466
C8	0.3514	0.7254	0.1184	0.4513	0.0833	0.1208	0.9330
C9	0.6319	0.9424	0.3403	0.8222	0.1090	0.1547	0.9103
C9i	0.7555	0.9825	0.4752	0.9252	0.1410	0.1902	0.8811
C9ii	0.3707	0.7261	0.1179	0.4310	0.0942	0.1402	0.9256
C9iii	0.2332	0.5843	0.0251	0.1600	0.0838	0.1326	0.9364
C10	0.3808	0.7631	0.1173	0.4332	0.0984	0.1443	0.9215
C10i*	0.0169	0.0569	0.0001	0.0012	0.0094	0.0166	0.9934
C10ii	0.0775	0.2479	0.0083	0.0621	0.0274	0.0432	0.9793
C12*	0.0003	0.0021	0	0	0.0002	0.0004	0.9998
C13	0.1031	0.3065	0.0022	0.0285	0.0472	0.0777	0.9659
C14	0.0832	0.2214	0	0.0002	0.0514	0.0915	0.9647
C14i	0.2460	0.5948	0.0467	0.2441	0.0736	0.1128	0.9429
C15*	0.0007	0.0050	0	0.0001	0.0004	0.0008	0.9997
C16	0.1460	0.3750	0.0357	0.1545	0.0427	0.0653	0.9668
C17	0.4761	0.8490	0.1955	0.6168	0.1019	0.1458	0.9168

C18*	0.0001	0.0001	0	0	0	0.0001	1.0000
C19**	0.0652	0.1921	0.0011	0.0127	0.0313	0.0521	0.9772
C20*	0.0464	0.1211	0	0	0.0313	0.0566	0.9786
C21*	0.0344	0.0773	0	0	0.0294	0.0557	0.9808
C22*	0.0001	0.0003	0	0	0.0001	0.0001	1.0000
C23*	0.0046	0.0110	0	0	0.0037	0.0074	0.9976
C24*	0	0	0	0	0	0	1.0000
C25*	0.0009	0.0044	0	0.0001	0.0010	0.0009	0.9996
C26	0.0552	0.1610	0.0002	0.0028	0.0310	0.0533	0.9781
C27*	0.0048	0.0128	0	0	0.0035	0.0063	0.9977
C28	0.4736	0.8668	0.1772	0.6132	0.1041	0.1495	0.9157
C29*	0.0091	0.0318	0	0.0002	0.0055	0.0095	0.9962
C30	0.0856	0.2418	0.0001	0.0019	0.0481	0.0860	0.9666
C31*	0.0226	0.0499	0	0	0.0197	0.0378	0.9871
C32*	0.0087	0.0211	0	0	0.0067	0.0120	0.9953
C33*	0.0474	0.1359	0.0001	0.0009	0.0285	0.0512	0.9801
C34	0.6678	0.9510	0.3850	0.8518	0.1186	0.1644	0.9012
DIAM1	0.1590	0.4313	0.0005	0.0119	0.0799	0.1345	0.9427
DIAM2	0.3488	0.7289	0.1228	0.4721	0.0779	0.1138	0.9376
HEIGHT1	0.1483	0.4266	0.0013	0.0275	0.0673	0.1130	0.9517
HEIGHT2	0.3485	0.7354	0.1043	0.4230	0.0839	0.1261	0.9337
LFAREA	0.2431	0.6442	0.0174	0.1712	0.0845	0.1323	0.9352
LFSHAP	0.0852	0.2400	0	0.0006	0.0475	0.0804	0.9658

Table S4. Candidate genes identified in the genomic windows with Posterior Inclusion Probability (PIP) ≥0.4.

Genes	Chromosome	Start Pos.	End Pos.	Traits	Category	*Arabidopsis thaliana* corresponding gene	Protein name	Description
Potri.001G005100	Chr01	339493	342618	C32	Flavonoid	AT3G13540.1	ATMYB5, MYB5	myb domain protein 5
Potri.001G007000	Chr01	493207	494619	C32	Flavonoid	AT1G15670.1		Galactose oxidase/kelch repeat superfamily protein. Negatively regulate phenylpropanoid biosynthesis by targeting the phenypropanoid biosynthesis enzyme phenylalanine ammonia-lyase.
Potri.003G138200	Chr03	15639785	15641810	C31	Flavonoid	AT4G01070.1	GT72B1, UGT72B1	UDP-Glycosyltransferase superfamily protein
Potri.003G138400	Chr03	15650072	15653385	C31	Flavonoid	AT5G42800.1	DFR, M318, TT3	dihydroflavonol 4-reductase
Potri.003G139600	Chr03	15733245	15738267	C31	Flavonoid	AT1G64390.1	AtGH9C2, GH9C2	glycosyl hydrolase 9C2
Potri.003G140900	Chr03	15813955	15817854	C31	Flavonoid	AT4G10960.1	UGE5	UDP-D-glucose/UDP-D-galactose 4-epimerase 5
Potri.006G190800	Chr06	20556974	20558623	C20	Flavonoid	AT2G42250.1	CYP712A1	cytochrome P450, family 712, subfamily A, polypeptide 1
Potri.006G191000	Chr06	20569291	20573186	C20	Flavonoid	AT5G06800.1		myb-like HTH transcriptional regulator family protein
Potri.011G060300	Chr11	5414925	5416493	C18, C23, C19_binary	Flavonoid	AT5G54010.1		Flavonoid 3-O-glucosyltransferas

Potri.011G061000	Chr11	5451888	5453414	C18, C23, C19_binary	Flavonoid	AT5G54010.1		Flavonoid 3-O-glucosyltransferas
Potri.011G079400	Chr11	7883530	7889818	C18, C21	Flavonoid	AT2G34410.1		O-acetyltransferase family protein
Potri.011G080000	Chr11	7934591	7935310	C18, C21	Flavonoid	AT1G29950.1		basic helix-loop-helix (bHLH) DNA-binding superfamily protein
Potri.012G138800	Chr12	15331801	15333280	C12, C15	Salicinoid	AT5G13930.1	ATCHS, CHS, TT4	Chalcone and stilbene synthase family protein
Potri.012G034100	Chr12	3047772	3049697	C19, C24, C25, C29, C31, C29_binary, C32_binary	Flavonoid	AT2G22590.1		UDP-Glycosyltransferase superfamily protein
Potri.012G035800	Chr12	3205152	3206537	C19, C24, C25, C29, C31, C29_binary, C32_binary	Flavonoid	AT3G16520.3	UGT88A1	UDP-glucosyl transferase 88A1
Potri.012G036000	Chr12	3225625	3227245	C19, C24, C25, C29, C31, C29_binary, C32_binary	Flavonoid	AT4G01070.1	GT72B1, UGT72B1	UDP-Glycosyltransferase superfamily protein
Potri.012G139300	Chr12	15374256	15378451	C12, C15	Salicinoid	AT4G00730.1	AHDP, ANL2	Homeobox-leucine zipper family protein. Involved in the accumulation of anthocyanin
Potri.012G140500	Chr12	15445809	15448186	C12, C15	Salicinoid	AT5G52260.1	AtMYB19, MYB19	myb domain protein 19
Potri.012G140700	Chr12	15454375	15457724	C12, C15	Salicinoid	AT3G61250.1	AtMYB17, MYB17	myb domain protein 17
Potri.013G146200	Chr13	15147780	15151940	C32	Flavonoid	AT2G20810.1	GAUT10, LGT4	Galacturonosyltransferase

Potri.013G148600	Chr13	15294938	15296422	C32	Flavonoid	AT4G21440.1	ATM4, ATMYB102, MYB102	MYB-like 102
Potri.013G149100	Chr13	15342945	15344193	C32	Flavonoid	AT3G23250.1	ATMYB15, ATY19, MYB15	myb domain protein 15
Potri.013G149200	Chr13	15353474	15356043	C32	Flavonoid	AT2G31180.1	ATMYB14, MYB14, MYB14AT	myb domain protein 14
Potri.015G002600	Chr15	162021	163539	C22, C27	Flavonoid	AT5G24520.1	ATTTG1, TTG, TTG1, URM23	Transducin/WD40 repeat-like superfamily protein. Affects dihydroflavonol 4-reductase gene expression
Potri.015G003100	Chr15	236720	239777	C22, C27	Flavonoid	AT5G54160.1	ATOMT1, OMT1	flavonol O-methyltransferase 1
Potri.015G010100	Chr15	673277	675584	C23, C31	Flavonoid	AT5G24318.1		O-Glycosyl hydrolases family 17 protein
Potri.018G131600	Chr18	15289123	15290383	C6	CA	AT2G41480.1		Peroxidase superfamily protein

Methods S1 RAD-seq data processing, reference-mapping and variant calling

We processed the reads from RAD-seq with several bioinformatic tools: first, we assigned them to each individual according to their unique barcode through the program fastq-multx (ea-utils; Aronesty, 2011), allowing one mismatch in the 15 bp including barcode and restriction site. FastQC 0.10.1 (Andrews, 2010) was used to check the quality of the data and low quality bases and reads were removed with condetri v.2.2 (Smeds & Künstner, 2011) using default parameters, except for the option -*lfrac* (maximum acceptable fraction of bases after quality trimming with quality scores lower than the threshold -*lq*), for which a value of 0.1 was chosen.
Good quality reads were aligned against the *P. trichocarpa* reference genome (Ptrichocarpa_210_v3.0; Tuskan *et al.*, 2006). For this purpose, we used Bowtie2 2.2.4 (Langmead & Salzberg, 2012) with "end-to-end" and "very sensitive" settings. Reads with mapping quality lower than 10 were discarded using samtools 1.2 (Li *et al.*, 2009) and read group information was added with picard tools 1.130 (http://broadinstitute.github.io/picard). The files for each individual were run through several tools in GATK 3.4.46 (DePristo *et al.*, 2011): TargetCreator and IndelRealigner to realign around indels, BaseRecalibrator to recalibrate base quality scores using the SNVs from Christe *et al.*(2016) as set of known SNVs, and UnifiedGenotyper for variant and genotype calling. This last step was run on the full dataset to obtain the data used in entropy (Gompert *et al.*, 2014), while for RASPberry it was limited to calling genotypes at SNVs from Christe *et al.*(2016) that were covered in all parental individuals, using the option EMIT_ALL_SITES.

Methods S2 Inference of local and genome-wide ancestry

We used the computer program RASPberry (Wegmann *et al.*, 2011)to estimate local ancestry for our Genome-Wide Association Study (GWAS) mapping population, following the rationale and general principles outlined in our recent poplar hybrid zone study by Christe *et al.* (2016), including improvements that were necessary primarily because of the lower sequencing coverage used here. Firstly, we incorporated genotyping error expected for RAD-seq in the RASPberry model by using an estimate of the per-allele error rate obtained with the software TIGER (Tools for Integrating Genotyping ERrors; https://bitbucket.org/wegmannlab/tiger) as input value for the

miscopying mutation parameter (0.0625). The complete procedure for estimating and correcting RAD-seq genotyping error is presented elsewhere (Bresadola *et al.*, 2019), along with a detailed description of the TIGER software. For the species' mutation rates we added this estimate to the previously available values, scaled by the size of the reference panel (0.00185 and 0.00349 for *P. alba* and *P. tremula*, respectively) as recommended (Wegmann *et al.*, 2011). To reflect differences in admixed individuals and reference panels compared to Christe *et al.*(2016), we re-estimated the remaining admixture parameters, namely the time since admixture and the ancestral recombination rates among the *P. alba* and *P. tremula* reference haplotypes. Towards this end, we maximized the likelihood across all individuals but arbitrarily limited to markers on chromosomes 7 and 11 to reduce computational burden. We thus optimized each parameter in turn using the following values: 100, 200, 300, 500, 750, 1000, 2000, 3500, 5000, 7000, 8500 and 10000 for the ancestral recombination rates; 2, 3, 5, 7, 10, 15, 20, 50, 75 and 100 for the number of generations since admixture; 0.01, 0.02, 0.03, 0.04, 0.05, 0.06, 0.07, 0.08, 0.09, 0.1, 0.12, 0.15, 0.17, 0.2, 0.22 and 0.25 for the miscopying rate.

Genome-wide ancestry (*q*) was used for several purposes in our study, e.g. to check the genomic composition of our admixture mapping GWAS panel (Fig. 1**a**), as prior information for estimating local ancestry in RASPberry, and to regress out *q* during polygenic modeling of quantitative traits with GEMMA (Zhou *et al.*, 2013; below, Methods S3). In general, we used *q* estimates from entropy (Gompert *et al.*, 2014) in our study. To account for genotyping errors, we ran entropy directly on genotype likelihoods, which we adjusted mathematically to incorporate estimated RAD-seq genotyping error following the procedure implemented in TIGER and described in (Bresadola *et al.*, 2019). Only when running RASPberry, we used an alternative approach, and estimated *q* with ADMIXTURE (Alexander *et al.*, 2009) based on the SNVs used for local ancestry inference. However, the two estimates of *q* were highly correlated (Pearson's $r = 0.9897$; p-value <2.2e-16).

Methods S3 Rationale for choice of plant traits measured in this study

In this study, we measured a range of traits that differ among the parental species and their hybrids. In particular, we focused on phytochemical traits (the abundances of phenylpropanoid secondary metabolites in leaves), leaf morphology and growth-related traits. This set of traits

was chosen because of its potential relationship with plant performance and fitness: phenylpropanoid molecules are involved in numerous important processes in plants, including structural support (cell wall fortification and wood formation), disease resistance, UV protection, and plant-animal mutualism (Dixon *et al.*, 2002; Chen *et al.*, 2009; Vogt, 2010; Caseys *et al.*, 2015). Moreover, the underlying genes and pathways are well known compared to most other traits in plants. Leaf area is associated with biomass gain in poplar (Rae *et al.*, 2004) and greater leaf size likely correlates with higher competitive ability (Rae *et al.*, 2006). Plant height is often used as an early fitness proxy in perennial plants (Younginger *et al.*, 2017), and trade-offs between growth and defense are of major interest in plant biology (Züst & Agrawal, 2017). All traits studied here represent ecologically relevant phenotypic trait differences between *P. alba* and *P. tremula*, thus they may be involved in reproductive isolation between species.

Methods S4 Admixture mapping with GEMMA: model choice and validation

We used GEMMA 0.94.1 (Zhou *et al.*, 2013) to scan the genome of admixed individuals for associations between ancestry segments and phenotypes (cf. admixture mapping). GEMMA offers the opportunity to choose between fitting a univariate linear mixed model (LMM), a multivariate linear mixed model (LMM) and a Bayesian sparse linear mixed model (BSLMM). We used BSLMM (option *-bslmm 1*) because it implements a polygenic approach, in which the effect of multiple loci on the phenotype is evaluated simultaneously, rather than a single locus at a time. Most of our modeling decisions and rationales are described in the main paper.

The kinship matrix calculated by GEMMA based on our input data accounted for the similarity at the level of genome-wide ancestry. Being a centered matrix, it showed the deviation of the genomic similarity between a pair of individuals from its mean value: *P. tremula*-like individuals deviated from this in a specific direction, while *P. alba*-like individuals deviated in the opposite direction (Fig. **S2**). In addition to genome-wide ancestry patterns, this matrix also captured genetic relationships within and among the open-pollinated families.

To evaluate the extent to which we came to similar conclusions about trait genetics with different models, we used multiple analysis options in GEMMA, including *-notsnp*. This option allowed us to directly use the phenotypic measurements (rather than residuals as described in the main paper) and to include the covariates in the file together with the input genotypic information (this

was possible because the *-notsnp* option disables the filter for minor allele frequency). We note that in *-notsnp* runs, the parameter estimates associated with genomic architecture (*PVE, PGE* and *n_gamma*) should be interpreted with caution, because they also include the effect of *q*, planting year and common garden location.

For each trait we performed 10 independent model runs, with 2 million burn-in steps and 10 million iterations. The burn-in steps were discarded and convergence was assessed visually by means of trace plots and comparing the consistency of the results across runs. To obtain the posterior distributions of the hyperparameters, we down-sampled the 10 chains, saving 1 value out of 10, and combined chains to obtain posterior distributions. Values of Posterior Inclusion Probability (PIP) per 0.5 Mb windows were calculated separately for the 10 runs and then averaged. Only for one trait (the flavonoid kaempferol-rutinoside-pentose - C21) a subset of the 10 chains suggested the presence of an alternative solution, providing different posterior distributions for the hyperparameters and different PIPs values. We therefore ran a total of 100 chains, to be able to evaluate the validity of the second solution and to calculate a reliable average when combining all chains.

For 12 phytochemical compounds, trait values (molecular abundances of specific secondary metabolites) were equal to zero in more than 10% of the individuals. In these cases we also ran the trait in a binary version, replacing with 1 all the observations larger than 0. We then used a binomial logistic regression to obtain the residuals to be used as phenotypic information in GEMMA. These runs were not taken into account to examine the posterior distributions of the hyperparameters, but only when selecting interesting genomic regions for selected traits.

To further evaluate the consistency of our results when using different analysis options, we also ran the univariate linear mixed model (LMM) in GEMMA (option *-lmm 2*), which provides a p-value deriving from a likelihood ratio test for each site.

Notes S1 Genomic windows highlighted by alternative modeling approaches in GEMMA

BSLMMs using the "-notsnp*" option:*To evaluate the extent to which our results were consistent among different analysis options in GEMMA, we ran the program also with the raw phenotypic data (not the residuals) and the *-notsnp* option. This resulted in highly congruent results. For traits C6, C10i, C12, C18, C19 (quantitative and binary) C20, C21, C27, C29 (quantitative and

binary), C31, C32 (quantitative) and C33, the windows exceeding the Posterior Inclusion Probability (PIP) threshold of 0.4 were exactly the same between GEMMA runs based on the residuals or directly on the measurements. For three traits (C15, C22 and C23), there was an additional window in the -*notsnp* run compared to the run on the residuals, while for C25 and C32 (binary) one window had PIP ≥0.4 in the run on the residuals, but none reached the threshold in the -*notsnp* run. Only for one trait (C24), the windows with high PIP emerging from the run on the residuals and from the -*notnsp* run were different, although nearby on chromosome 12 (window between 3 and 3.5 Mb and between 4 and 4.5 Mb in the run on the residuals and in the -*notsnp* run, respectively).

Linear Mixed Models (LMM):The single-SNV analysis (LMM), when compared to BSLMM, highlighted a significant association only for one trait (C24) if using $5x10^{-8}$ as threshold for the significance of the p-value, a common practice in GWAS applications (Barsh *et al.*, 2012). Out of the ten top-ranking sites for this trait, four are located in the window highlighted by BSLMM, while the remaining six are found in a flanking window downstream. Even considering a less stringent p-value of 10^{-7}, we would have been able to identify associations only for three additional traits: C12, C15 and C18. The ten sites with the lowest p-value for C12, C15 and C18 are located in the windows with PIP ≥0.4 we selected based on the BSLMM results. In general, LMM identified a much smaller number of significant associations since independently testing each site for association is less powerful than applying polygenic approaches.

Notes S2 Additional information on candidate genes

This admixture mapping study revealed several candidate gene-trait associations with potential relevance for functional plant biology and evolutionary genetics. The best candidate genes are described below while other candidate genes with potential relation to the traits are listed in Table S3.

The flavonoid isorhamnetin-glycuronide (C32) showed association with the first window on chromosome 1 (0.5 - 1.0 Mb), containing the gene Potri.001G005100, which encodes a MYB transcription factor (MYB5 in *Arabidopsis thaliana*). This compound was also associated with a window on chromosome 13 (15.0 - 15.5 Mb), which conspicuously contains two additional MYB genes (Potri.013G149100 - MYB14 in *A. thaliana*, and Potri.013G149200 - MYB15 in *A.*

thaliana), a finding discussed in more depth in the main paper. These windows also contain other candidates, the gene Potri.001G007000 (chromosome 1) encodes a Kelch repeat F-box that regulates phenylpropanoid biosynthesis in *A. thaliana* (Zhang *et al.*, 2013), while Potri.013G146200 (chromosome 13) encodes a protein with a putative galacturonosyl transferase activity. In isorhamnetin-glycuronide, the sugar is either in glucuronide or galacturonide form making this gene a likely candidate.

The interval between 5 and 5.5 Mb on chromosome 11 was associated with three quercetins linked to glucose-based sugars (quercetin rutinoside-pentose C18; quercetin-3-O-rutinoside C23 and quercetin-glucuronide-pentose binary C19). This genomic region hosts two genes (Potri.011G060300 and Potri.011G061000) whose orthologs in *A. thaliana* are known to encode flavonoid 3-O-glucosyl transferases acting as catalysts for the transfer of glycosyl groups, which represent important chemical modifications to flavonoid core molecules (Yonekura-Sakakibara & Saito, 2014). In the *P. trichocarpa*genome (annotation v3.0), these genes are annotated as Anthocyanidin 3-O-glucoside 2''-O-glucosyl transferases. This study suggests that their activity may extend to the flavonols in *Populus*.

Similarly, the window between 3 and 3.5 Mb on chromosome 12 was significantly associated with several traits, in particular the flavonoids quercetin-glucuronide-pentose (C19), quercetin-3-O-glucuronide (C24), quercetin-3-O-glucoside (C25), kaempferol-glycuronide (C29 and binary C29), isorhamnetin-glycoside (C31), isorhamnetin-glycuronide (binary C32). This region contains three genes (Potri.012G034100, Potri.012G035800 and Potri.012G036000) annotated in *A. thaliana* as UDP-glycosyl transferases. Potri.012G036000 has further been characterized as a chalcone 4'-O-glucosyl transferase in *P. trichocarpa* and according to Phytozome (https://phytozome.jgi.doe.gov) its expression profile shows a correlation coefficient of 0.953 with that of flavonol-synthase 1 (Potri.004G139700), a key enzyme of the flavonoid pathway.

Among other noteworthy genetic associations, the window of interest on chromosome 15 (500001 bp to 1 Mb) exhibited association with the flavonols quercetin-3-O-rutinoside (C23) and isorhamnetin-glycoside (C31). In this window, the gene Potri.015G010100 was annotated as encoding a O-glycosyl hydrolase family 17 protein, that reduces the complexity of sugar moieties of phenylpropanoids. On the other hand, the first 0.5 Mb in chromosome 15 were associated with two isorhamnetins (the flavonoids isorhamnetin rutinoside-pentose (C22) and isorhamnetin-3-O-rutinoside C27): this window contains the gene Potri.015G003100, whose

ortholog in *A. thaliana* encodes a flavonol O-methyltransferase 1 that converts quercetins into isorhamnetins (potentially converts C18 to C22 and C23 to C27).

Isorhamnetin-glycoside (C31) showed significant association with the window located between 15.5 and 16 Mb on chromosome 3. This window hosts the gene Potri.003G138200, also annotated as UDP-glycosyl transferase.

Finally, the window between 15 and 15.5 Mb on chromosome 12 exhibited a significant association with the salicinoids HCH-Salicortin (C12) and HCH-tremulacin (C15). One candidate gene of special interest in this region is Potri.012G138800, encoding a chalcone-synthase (CHS). From the viewpoint of functional plant biology, the chalcone-synthase (CHS) (Potri.012G138800) is of particular interest. While this enzyme is essential to the flavonoid pathway due to its basal role, this gene would *not* normally be expected to be associated with salicinoid biosynthesis. The *Populus* genome contains at least six CHS genes (Tsai *et al.*, 2006). The role of CHS in the flavonoid pathway is the conversion of coumaroyl-CoA and malonyl-CoA into chalcone. In the salicinoid pathway, the polyketide synthase activity of the enzyme may act directly on benzoyl-CoA, which has recently been put forward as a potential precursor of this group of compounds (Babst *et al.*, 2010). The expression of this gene is correlated with that of many other genes (36 in total, according to Phytozome data) not involved in the biosynthesis of flavonoids, suggesting that this gene is indeed part of a different metabolic network. Knowledge regarding the biosynthetic pathway of salicinoids remains very poor. Chedgy *et al.*(2015) reported the functional characterization of two genes encoding acyl transferases and predicted to produce the secondary metabolites putatively involved in the benzenoid metabolism. Their findings are consistent with a potential role of these enzymes in the salicinoid pathway, but need to be confirmed by direct functional tests *in vivo*. To our knowledge, the CHS gene we identified is the first potential candidate gene put forward for the biosynthesis of salicinoids through a GWAS.

We note that two of the candidate genes described in this study were recently identified as having undergone adaptive protein evolution in one or both of these two hybridizing species (Christe *et al.*, 2017). Both genes were identified in genomic windows associated with isorhamnetin-glycuronide (C32). The galacturonosyl transferase found on chromosome 13 was found to be under positive selection in both *P. alba* and *P. tremula*, while MYB5 (chromosome 1) was affected by positive selection in *P. tremula* only.

References

Alexander DH, Novembre J, Lange K. **2009**. Fast model-based estimation of ancestry in unrelated individuals. *Genome Research* **19**: 1655–1664.

Andrews S. **2010**. *FastQC: a quality control tool for high throughput sequence data*. Available online at: http://www.bioinformatics.babraham.ac.uk/projects/fastqc.

Aronesty E. **2011**. ea-utils: command-line tools for processing biological sequencing data. *Durham, NC: Expression Analysis*.

Babst BA, Harding SA, Tsai C-J. **2010**. Biosynthesis of phenolic glycosides from phenylpropanoid and benzenoid precursors in *Populus*. *Journal of Chemical Ecology* **36**: 286–297.

Barsh GS, Copenhaver GP, Gibson G, Williams SM. **2012**. Guidelines for genome-wide association studies. *PLOS Genetics* **8**: e1002812.

Bresadola L, Link V, Buerkle CA, Lexer C, Wegmann D. **2019**. Estimating and accounting for genotyping errors in RAD-seq experiments. *bioRxiv*http://dx.doi.org/10.1101/587428.

Caseys C, Stritt C, Glauser G, Blanchard T, Lexer C. **2015**. Effects of hybridization and evolutionary constraints on secondary metabolites: the genetic architecture of phenylpropanoids in European *Populus* species. *PLOS ONE* **10**: 1–23.

Chedgy RJ, Köllner TG, Constabel CP. **2015**. Functional characterization of two acyltransferases from *Populus trichocarpa* capable of synthesizing benzyl benzoate and salicyl benzoate, potential intermediates in salicinoid phenolic glycoside biosynthesis. *Phytochemistry* **113**: 149–159.

Chen F, Liu C-J, Tschaplinski TJ, Zhao N. **2009**. Genomics of secondary metabolism in *Populus*: interactions with biotic and abiotic environments. *Critical Reviews in Plant Sciences* **28**: 375–392.

Christe C, Stölting KN, Bresadola L, Fussi B, Heinze B, Wegmann D, Lexer C. **2016**. Selection against recombinant hybrids maintains reproductive isolation in hybridizing *Populus* species despite F1 fertility and recurrent gene flow. *Molecular Ecology* **25**: 2482–2498.

Christe C, Stölting KN, Paris M, Fraïsse C, Bierne N, Lexer C. **2017**. Adaptive evolution and segregating load contribute to the genomic landscape of divergence in two tree species connected by episodic gene flow. *Molecular Ecology* **26**: 59–76.

DePristo MA, Banks E, Poplin R, Garimella K V, Maguire JR, Hartl C, Philippakis AA, del Angel G, Rivas MA, Hanna M, *et al.* 2011. A framework for variation discovery and genotyping using next-generation DNA sequencing data. *Nature Genetics* **43**: 491-498.

Dixon RA, Achnine L, Kota P, Liu CJ, Reddy MSS, Wang L. **2002**. The phenylpropanoid pathway and plant defence - A genomics perspective. *Molecular Plant Pathology* **3**: 371–390.

Gompert Z, Lucas LK, Buerkle CA, Forister ML, Fordyce JA, Nice CC. **2014**. Admixture and the organization of genetic diversity in a butterfly species complex revealed through common and rare genetic variants. *Molecular Ecology* **23**: 4555–4573.

Langmead B, Salzberg SL. **2012**. Fast gapped-read alignment with Bowtie 2. *Nature Methods* **9**: 357-359.

Li H, Handsaker B, Wysoker A, Fennell T, Ruan J, Homer N, Marth G, Abecasis G, Durbin R, Subgroup 1000 Genome Project Data Processing. **2009**. The Sequence Alignment/Map format and SAMtools. *Bioinformatics* **25**: 2078–2079.

Rae AM, Ferris R, Tallis MJ, Taylor G. **2006**. Elucidating genomic regions determining enhanced leaf growth and delayed senescence in elevated CO2. *Plant, Cell and Environment* **29**: 1730–1741.

Rae AM, Robinson KM, Street NR, Taylor G. **2004**. Morphological and physiological traits influencing biomass productivity in short-rotation coppice poplar. *Canadian Journal of Forest Research* **34**: 1488–1498.

Smeds L, Künstner A. **2011**. ConDeTri - A content dependent read trimmer for Illumina data. *PLoS ONE* **6**: 1–6.

Tsai C-J, Harding SA, Tschaplinski TJ, Lindroth RL, Yuan Y. **2006**. Genome-wide analysis of the structural genes regulating defense phenylpropanoid metabolism in *Populus*. *New Phytologist* **172**: 47–62.

Tuskan GA, DiFazio S, Jansson S, Bohlmann J, Grigoriev I, Hellsten U, Putnam N, Ralph S, Rombauts S, Salamov A, *et al.*2006. The genome of black cottonwood, *Populus trichocarpa* (Torr. & Gray). *Science* **313**: 1596–1604.

Vogt T. **2010**. Phenylpropanoid biosynthesis. *Molecular Plant* **3**: 2–20.

Wegmann D, Kessner DE, Veeramah KR, Mathias RA, Nicolae DL, Yanek LR, Sun Y V, Torgerson DG, Rafaels N, Mosley T, *et al.* 2011. Recombination rates in admixed individuals identified by ancestry-based inference. *Nature Genetics* **43**: 847-853.

Yonekura-Sakakibara K, Saito K. **2014**. Function, structure, and evolution of flavonoid glycosyltransferases in plants. In: Romani A, Lattanzio V, Quideau S, eds. *Recent advances in polyphenol research*. John Wiley & Sons, **4**: 61–82.

Younginger BS, Sirová D, Cruzan MB, Ballhorn DJ. **2017**. Is biomass a reliable estimate of plant fitness? *Applications in Plant Sciences* **5**: 1600094.

Zhang X, Gou M, Liu C-J. **2013**. *Arabidopsis* Kelch repeat F-Box proteins regulate phenylpropanoid biosynthesis via controlling the turnover of phenylalanine ammonia-lyase. *The Plant Cell* **25**: 4994–5010.

Zhou X, Carbonetto P, Stephens M. **2013**. Polygenic modeling with Bayesian sparse linear mixed models. *PLOS Genetics* **9**: 1–14.

Züst T, Agrawal AA. **2017**. Trade-offs between plant growth and defense against insect herbivory: an emerging mechanistic synthesis. *Annual Review of Plant Biology* **68**: 513–534.
